# Ultrafast CTCF dynamics control cohesin barrier function

**DOI:** 10.1101/2025.11.25.690553

**Published:** 2025-11-29

**Authors:** Sergei Rudnizky, Peter J Murray, Emily W. Sørensen, Theo J. R. Koenig, Sushil Pangeni, Raquel Merino-Urteaga, Hemani Chhabra, Laura Caccianini, Iain F. Davidson, Manuel Osorio-Valeriano, Paul W Hook, Paul Meneses, Jingzhou Hao, Jasmin S. Zarb, Nikos S Hatzakis, Winston Timp, Lucas Farnung, Seychelle M. Vos, Jan-Michael Peters, Aleksei Aksimentiev, Taekjip Ha

## Abstract

CCCTC-binding transcription factor (CTCF) and the cohesin complex shape the genome into loops and topologically associating domains (TADs), yet the mechanisms linking CTCF dynamic behavior to its function as a cohesin barrier remain unclear. Using integrated experimental and computational approaches, we demonstrate that individual CTCF-DNA complexes are intrinsically mobile over distances and timescales compatible with cohesin capture during loop extrusion, driven by the dynamic rearrangements of zinc finger (ZF) domains. Genome-wide single-molecule accessibility and sequencing analyses further reveal that these dynamics occur in cells. This intrinsic flexibility is modulated by DNA sequence, methylation, and nucleosome positioning, and enables a previously uncharacterized single-stranded DNA binding state. Through biochemical dissection of cohesin components, we find that a negative regulator of loop extrusion, PDS5, remodels ZF dynamics and imparts exceptional mechanical stability to CTCF on DNA. Our findings reveal that the conformational dynamics of a transcription factor constitute a fundamental regulatory property that bridges base pair-scale molecular motions to megabase-scale genome organization, offering a kinetic explanation for how CTCF establishes and regulates TAD boundaries.

### INTRODUCTION

Eukaryotic genomes are organized into chromatin loops, which constrain or facilitate chromosomal contacts, defining regulatory units[1–3,4]. In mammals, CTCF plays a central role in shaping these loops by acting as a chromatin barrier[5–9] to cohesin-mediated DNA extrusion[10,11], facilitating the formation of TADs[1,3,8,9,12]. The proteins involved in TAD formation are broadly conserved across vertebrates[13], and their dysregulation has been linked to a range of diseases, including developmental disorders[14], cohesinopathies[15], and cancer[16,17].

CTCF[18] is an eleven ZF transcription factor (TF) that wraps around DNA through the major groove of its binding motif[19–22]. This binding motif is enriched at TAD boundaries[1,4], where CTCF is required for cohesin accumulation[7,12,23] and boundary formation. CTCF has minute-scale residence time on chromatin[24,25], with the largest contribution coming from its high-affinity, central ZFs, which bind at the least variable core region of the binding motif[25,26]. The CTCF ZFs are flanked by N- and C-terminal intrinsically disordered regions (IDRs)[27], which contribute to its functions as an asymmetric, orientation-dependent roadblock[22,28]. The N-terminal IDR of CTCF contains binding sites for cohesin[29,30] and is required for proper TAD insulation and loop anchoring at CTCF sites[29–32]. In contrast, cohesin is more likely to bypass CTCF when it encounters the C-terminal IDR first[22,28]. In addition to the N-terminal IDR, the N-terminal ZFs are necessary for maximum stalling efficiency[30,32]. The precise mechanistic basis of the interplay between IDR and ZF contributions to CTCF's boundary element function remains unknown[33].

For cohesin-mediated loop extrusion to occur, its ATPase-activating NIPBL subunit is required[10]. This subunit functionally competes with the mutually exclusive, non-extruding, PDS5 subunit[34,35]. Despite NIPBL's essential role in extrusion, unlike other cohesin subunits, such as STAG1/STAG2[29,30,36] and PDS5A/B[30], NIPBL does not appear to interact directly with CTCF[22]. In mammalian cells, these CTCF-cohesin encounters occur in the dynamic chromatin environment, where CTCF occupancy and activity are modulated by motif sequence variation[37], CpG methylation[16,17,19], nucleosome positioning[38], transcription[39], replication[40], and other processes[41]. The mechanisms by which chromatin context and cohesin subunit composition modulate CTCF's insulator function remain unclear[33].

In this dynamic environment, cohesin capture by CTCF is constrained to a tight spatiotemporal window. A correctly oriented CTCF molecule must intercept a single cohesin complex translocating on DNA at 1-3 kb/s[10,42]. With an N-terminal IDR capture radius of 5 nm[27], CTCF only has ~15-50 ms to interact with the extruding cohesin[33], imperfectly stalling the complex in a tension-dependent manner[28,43]. The recapitulation of orientation-dependent, partial stalling *in vitro*[28] suggests these properties are intrinsic, motivating a mechanistic dissection of how a single CTCF-DNA complex is capable of rapid capture, yet often permits bypass.

Here, we measured CTCF-DNA interactions at the distances, forces, and timescales relevant to cohesin capture in cells. By probing individual CTCF-DNA complexes at high spatiotemporal resolution, we resolve their domain-level interactions and dynamics within a chromatin context and in the presence of cohesin and its auxiliary subunits. Our work identified the kinetic principles that allow CTCF to modulate cohesin activity and establish chromatin loop boundaries. In this way, CTCF functions across orders of magnitudes, bridging base-pair-scale dynamics to megabase-scale genome organization.

### RESULTS

#### CTCF ZF–DNA contacts are asymmetric

CTCF stalls cohesin more effectively when approached from its N-terminal side, demonstrating an intrinsic polarity to the barrier[28]. Because asymmetric protein-DNA interactions are key features of other polar barriers to translocases[44], we asked whether contacts between CTCF's ZF array[19,20] and its binding motif are similarly directional.

Using a high-resolution optical tweezers unzipping assay,[45–47] we measured the position and strength of local protein-DNA contacts within single human CTCF-DNA complexes. We unzipped these complexes on the consensus motif[19,26] or the oncogenic *PDGFRA* insulator[9] (Fig. 1A-B). While unzipping, when the fork encountered protein-DNA interactions, an increase in force ("rupture force") above the unbound DNA baseline was observed. 95% of these rupture events were at the expected motif position (Fig. 1A-C, S1A-D, Materials and Methods). Consistent with CTCF's established role as a directional barrier[2,3,28–30,37,48], rupture forces were asymmetric with respect to unzipping orientation. For both DNA motifs, the force required to disrupt protein-DNA interactions was higher when probed from the N-terminal side (18.6 ± 0.3 pN for *PDGFRA*, 20.1 ± 0.2 pN for consensus) than from the C-terminal side (14.3 ± 0.1 pN for *PDGFRA*, 18.1 ± 0.2 pN for consensus) (Fig. 1D-E). These asymmetric CTCF-DNA interactions appeared to be conserved in mouse CTCF (Fig. S1E-H). Furthermore, the *PDGFRA* sequence had weaker CTCF-DNA contacts from both orientations than the consensus motif, consistent with previous observations of reduced affinity upon deviation from the consensus[25] (Fig. S1A).

To determine whether the IDRs contribute to the measured resistance, we truncated the N-terminal (ΔN CTCF) or C-terminal IDR (ΔC CTCF). Neither truncation significantly altered rupture forces, indicating that the probed protein-DNA interactions were dominated by the CTCF ZFs (Fig. 1E, Fig. S1I). Corresponding force-weighted dwell-time maps localized interactions to the ~30-40 bp footprint of the ZF array (Fig. 1F), with stronger interactions on the N-terminal side than on the C-terminal side, with no substantial effect of truncations (Fig. S1J). Together, these data suggest that CTCF's ZF

array interacts with DNA asymmetrically, with the N-terminal side binding DNA more strongly than its C-terminal side.

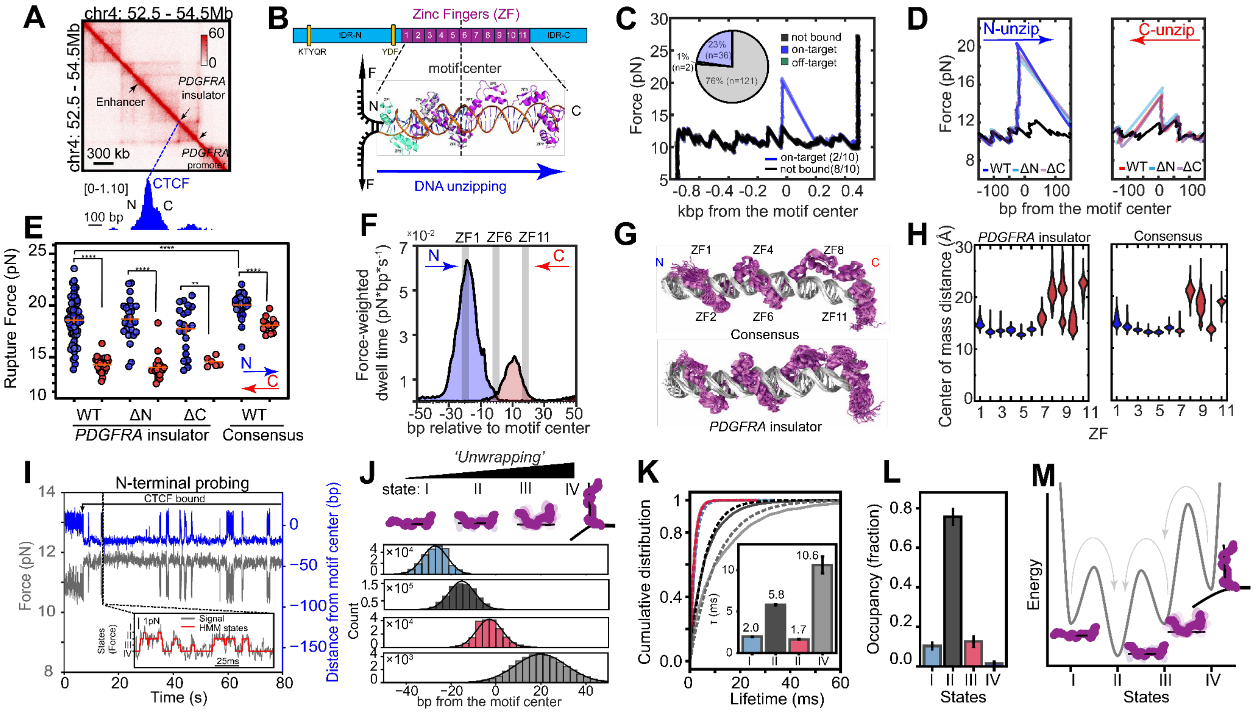

**Fig. 1: CTCF ZFs are intrinsically mobile mechanical barriers. (A)** A Hi-C map (top) and CTCF CUT&RUN data (bottom) of the *PDGFRA* locus in HEK293T cells. Hi-C data was obtained from Marin-Gonzalez et al[49]. The locations of the insulator, corresponding promoter, and corresponding enhancer are labeled and highlighted with arrows. **(B)** Above: a to-scale domain map of the CTCF protein depicting its N-terminal IDR, 11 ZF domains, C-terminal IDR, and cohesin-interacting KTYQR and YDF motifs[29,30,36]. Below: a schematic representation of DNA unzipping while encountering an N-terminally oriented CTCF-bound complex. The depicted CTCF molecule is overlapping, partial crystal structures obtained from Yang et al[20]. **(C)** A representative force-extension plot of a DNA construct containing the consensus motif being repetitively unzipped and rezipped from the N-terminal orientation. All cycles are overlaid, 8 of which were unbound cycles (black) and 2 of which were specifically bound by CTCF (blue). The inset pie chart shows counts of cycles that were unbound (gray), specifically bound (blue), or nonspecifically bound (green) by CTCF in 19 independent cyclical unzipping traces containing a total of 159 cycles. **(D)** Representative force-extension traces of the *PDGFRA* motif from the N- (left) and C-terminal (right) orientations with WT (blue for N-terminal, red for C-terminal), ΔN (cyan), and ΔC (purple) CTCF. **(E)** Single-molecule rupture forces for CTCF-DNA interactions on the *PDGFRA* or consensus motif for WT, ΔN, and ΔC CTCF from the N-terminal (blue) and C-terminal (red) orientations. The mean rupture forces for the *PDGFRA* motif were 18.6 ± 0.3 pN (n=57), 18.8 ± 0.4 (n=23), 17.7 ± 0.5 (n=21) for WT, ΔN, and ΔC, respectively, from the N-terminal orientation and 14.3 ± 0.1 (n=31), 14.0 ± 0.3 (n=19), 14.5 ± 0.2 (n=6) for WT, ΔN, and ΔC, respectively, from the C-terminal orientation. The mean rupture forces for WT CTCF on the consensus motif were 20.1 ± 0.2 pN (n=34), 18.1 ± 0.2 (n=14) for the N- and C-terminal orientation, respectively. Data are mean ± standard error. Using a 1-D, two-state Kolmogorov-Smirnov (K-S) test, significance is indicated as follows: **p<0.01, ****p<0.0001. **(F)** Force-weighted dwell time map of CTCF bound to the *PDGFRA* motif as a function of bp position. Interactions probed from the C-terminal orientation are shown in red, while interactions probed from the N-terminal orientation are shown in blue. The locations of ZFs are overlaid as gray bars, assuming each ZF binds a single DNA triplet.[19,20] **(G)** Multiple overlaid snapshots (every 40 ns) of CTCF bound to its consensus motif (top) or the *PDGFRA* sequence (bottom) sampled by 1 μs molecular dynamics (MD) simulations. ZF domains are numbered. Corresponding pLDDT and PAE plots for the AlphaFold 3 initial condition can be found in Fig. S2. **(H)** Simulated distribution of distance between the center of mass of each ZF and its 3 nearest DNA bps for each ZF in the *PDGFRA* (left) and consensus (right) sequences. N-terminal ZFs (1-6) are shown in blue and C-terminal ZFs (7-11) are shown in red. A second replicate of each is shown in Fig. S2E. **(I)** A representative trace of *PDGFRA* motif fluctuations, in force (gray) and the corresponding unzipping fork location as base pairs from motif center (blue), with two distinct regimes, unbound and CTCF-bound, as indicated by the black arrow. The inset shows a representative zoomed in region of ms-scale force fluctuations fit with a 4 state HMM mode. **(J)** Each state's mapped base pair location constructed from 4 representative replicates of CTCF-*PDGFRA* dynamics as four gaussians (states I, II, III, and IV, from top to bottom). Gaussian fits (black lines) provide mean ± standard deviation for each state: -27.1 ± 5.5 (n = 234,310), -15.5 ± 5.7 (n = 772,942), -3.0 ± 5.7 (n = 200,878), and 20.1 ± 11.4 (n = 41,865) for states I-IV, respectively. Hypothesized corresponding conformations illustrated as cartoons. **(K)** Measured lifetimes of each state for the *PDGFRA* motif each fit with a single exponential function to determine state lifetimes: 1.98 ± 0.03 ms for state I, 5.81 ± 0.06 ms for state II, 1.65 ± 0.02 ms for state III, and 10.63 ± 1.00 ms for state IV, across 10 replicates (Table S1-2). **(L)** Occupancy (fraction of time spent) in each state measured from the N-terminus of the *PDGFRA* motif. Error bars of occupancy represent the standard deviation between replicates (Table S2). **(M)** An energy landscape[50] (Materials and Methods) of four conformational states of CTCF on the *PDGFRA* motif, with a cartoon showing different numbers of DNA-engaged CTCF ZFs across the states.

### CTCF ZFs are intrinsically mobile and rapidly sample multiple conformations

To assess how individual ZFs contribute to orientation- and sequence-dependent interactions, we performed all-atom MD simulations with femtosecond time-steps, starting from AlphaFold 3[51]-predicted structures of IDR-truncated CTCF (Fig. 1G). In agreement with unzipping results, for both CTCF motifs examined, C-terminal ZFs exhibited greater positional fluctuation and less hydrogen bonding than N-terminal ZFs, consistent with a weaker association to DNA (Fig. 1E, G-H, Fig. 2S). Additionally, relative to the consensus motif, the *PDGFRA* insulator exhibited greater ZF mobility (Fig. 1H, Fig. S2). This deviation was most prominently seen at the ZF7 contact region, which has the greatest divergence from motif consensus (Fig. 1H, Fig. S1A).

To experimentally test whether the high ZF domain mobility observed in MD simulations could be indicative of larger-scale conformational exploration at physiological, ms-timescales, we used optical tweezers unzipping to probe CTCF dynamics. By holding the unzipping fork at the CTCF binding site, thermal fluctuations in the absence of CTCF caused the DNA to stochastically open and close, manifesting as low and high force states, respectively[47,52] (Fig. S3A-C). In the presence of CTCF in solution, the high force state was stabilized, indicating duplex clamping by CTCF binding (Fig. S3B). In most binding events, CTCF remained bound throughout the 1-5 min acquisition (Fig. S3C, Table S1). Unexpectedly, transient fluctuations were observed, which have not been observed for other TFs probed in a similar assay[47,52,53] (Fig. 1I, S3C). These fluctuations persisted in the subsequent absence of free CTCF, indicating that we probed conformational dynamics of a single bound CTCF (Fig. S3B), not dissociation and rebinding (Fig. S3D).

Statistical analysis of fluctuations revealed that, unlike CTCF-free DNA with two states, CTCF-bound DNA was best described by four states (Fig. 1I-M, S4-6). These dynamics were force-independent in the examined regime, suggesting they were an intrinsic property of the complex (Fig. 1I-M, Fig. S7, Materials and Methods)[52]. Mapping the observed states to the motif (Fig. 1J, S4A) revealed, as previously proposed,[25] ZFs moving in blocks - early (N-terminal), middle (core), and late (C-terminal) - progressing almost exclusively in a sequential manner (state I ↔ state II ↔ state III ↔ state IV) (Fig. S8-12). In state I, all ZFs were bound; in state II, only the early ZFs were dissociated; in state III, only the late ZFs remained bound; and in state IV, all ZFs were dissociated (Fig. 1J, S8). This interpretation is supported by the observation that the CTCF-bound state IV was aligned with the unbound DNA open state (Fig. S4). The sequential and cooperative nature of these ZF association and dissociation events suggest a model where ZFs, which are typically “snaked” through the major groove[19–22], undergo spontaneous, reversible unwrapping and rewrapping around the DNA.

To assess whether these conformations persist on a timescale relevant to influence cohesin passage, we extracted the lifetimes and occupancies of each state. All states exhibited millisecond-scale lifetimes that remained stable over time, consistent with steady-state dynamics (Fig. 1K, Fig. S13, Table S2). For the *PDGFRA* motif, states II and IV were the longest-lived (5.8 ms and 10.6 ms lifetime, respectively), but state II dominated the landscape due to its high prevalence (76% occupancy), while state IV was rarely visited (1%) (Fig. 1L-M, S14-16, Table S2). ΔN CTCF largely preserved WT CTCF-*PDGFRA* dynamics, with no statistically significant state occupancy changes (Fig. 2A, S9-12,14-16), suggesting ZF-DNA interactions dominate the observed behavior. Notably, these lifetimes were on the same order of magnitude as cohesin’s estimated ~15-50 ms passage time through the CTCF footprint[10,11,22,28]. Given that CTCF stalls cohesin *in vitro* with about 50-90% efficiency, depending on DNA tension[28], we hypothesized that the most prevalent, partially-unwrapped state II constitutes a “cohesin-compatible” barrier conformation in kinetic competition with cohesin-incompatible conformations.

### Chromatin context modulates CTCF’s conformational dynamics

Having characterized CTCF-DNA dynamics, we next examined how local chromatin features, known to regulate TAD boundaries[17,37,54], would modulate this landscape by testing the effect of motif sequence, CpG methylation, and nearby nucleosome presence on state populations and transition rates.

Compared to *PDGFRA*, the consensus motif showed higher state II occupancy (76% for *PDGFRA,* 82% for consensus), and slower transition rates out of state II (Fig. 2A, S12,

S14, S15-17, Table S2), indicating a shift towards the partially-unwrapped conformation and away from the fully-wrapped conformation. Counterintuitively, the high affinity consensus motif was not associated with more ZFs being bound, but rather with an enrichment of the partially-unwrapped conformation. Because consensus-like sequences are enriched at constitutive TAD boundaries[37,54], our observation supports the model that the partially unwrapped state is cohesin-compatible.

Because CpG methylation is broadly associated with reduced CTCF binding[17], we asked how methylation reshapes the dynamic landscape in the disease-relevant context of the *PDGFRA* insulator. Methylating a regulatory CpG within the ZF4 triplet of the *PDGFRA* motif*,* which leads to dysfunction of CTCF boundary element[16], did not abolish CTCF binding in our unzipping assay (Fig. S1A, S18A-C), but caused state II occupancy to fall from 76% to 52% (Fig. 2A, S14, S16-17, Table S2) and state III occupancy to increase from 13% to 47%. This result was consistent with the expectation that methylation would destabilize key cytosine contacts in the central ZFs, promoting their unwrapping and pushing the system towards the highly-unwrapped state III[16,19,37].

At TAD boundaries, CTCF sites reside in nucleosome-free regions flanked by well-positioned nucleosomes[38,55,56]. To faithfully recapitulate this context *in vitro,* we performed MNase-seq and CTCF CUT&RUN in HEK293T cells examining the *PDGFRA* locus (Fig. 2B) and genome-wide signal (Fig. S18D). Informed by this data, we positioned a nucleosome, using the Widom 601 sequence[57], at a physiological distance from the N-terminal side of the CTCF motif in our unzipping construct. In contrast to prior experiments, this scheme necessitated C-terminal probing. In the absence of a nucleosome, C-terminal ZFs exhibited greater unwrapping than N-terminal ZFs (state II: 76% N-terminal, 46% C-terminal; state IV: 1% N-terminal, 17% C-terminal), consistent with lower rupture forces and MD-predicted higher mobility of C-terminal ZFs (Fig. 1E,H, S2, S14, S16-17, Table S2). Nucleosome presence shifted the landscape toward a more frequently wrapped profile (state II: 69%, state IV: 5%) without detectable changes in rupture forces (Fig. 2A, 2C-E, S14, S16-17, S18E-I). Therefore, a well-positioned N-terminal nucleosome biases CTCF dynamics from highly-unwrapped states towards a partially-unwrapped state. This raises the possibility that chromatin architecture can tune CTCF conformation, and thus, possibly boundary strength.

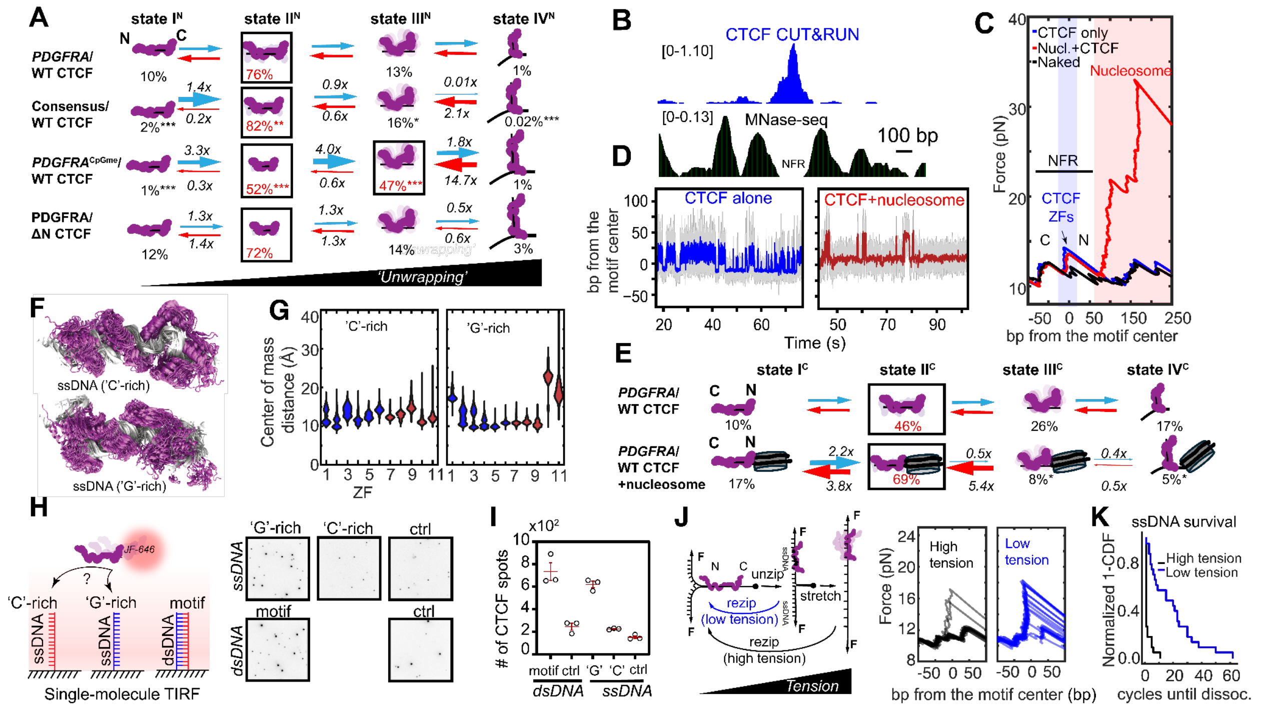

**Fig. 2: Chromatin context and transactions modulate CTCF conformations. (A)** Transition rates of CTCF state dynamics probed from the N-terminal orientation for, from top to bottom: *PDGFRA* with WT CTCF, consensus with WT CTCF, CpG methylated *PDGFRA* with WT CTCF, and *PDGFRA* with ΔN CTCF. Black boxes indicate the state(s) with the highest occupancy, or occupancies, with a percentage written in red. Other occupancy percentages are written in black. All transition rates and occupancies can be found in Figure S12, Figure S14, and Table S2. P-values are calculated with a two-tailed Mann-Whitney test relative to N-terminal WT CTCF bound to *PDGFRA* and can be found in Table S2, with significance indicated as *p<0.05,**p<0.01, ***p<0.001. **(B)** CUT&RUN for CTCF (above) and MNase-seq (below) at the *PDGFRA* motif in HEK293T cells. **(C)** A representative unzipping trace of CTCF on the *PDGFRA* motif probed from the C-terminal orientation with (red) and without (blue) an N-terminally-orientated, reconstituted, Widom 601 nucleosome. **(D)** Representative CTCF-*PDGFRA* fluctuations probed from the C-terminal orientation with (right, red) or without (left, blue) an N-terminal nucleosome. **(E)** Transition rates of CTCF state dynamics on the *PDGFRA* motif probed from the C-terminal orientation with (bottom) or without (top) an N-terminally-orientated nucleosome. Black boxes indicate the highest occupancy state, with a percentage written in red. Other occupancy percentages are written in black. All transition rates and occupancies can be found in Figure S12, Figure S14, and Table S2. P-values are calculated with a two-tailed Mann-Whitney test relative to C-terminal WT CTCF bound to *PDGFRA* and can be found in Table S2, with significance indicated as *p<0.05. **(F)** Multiple overlaid snapshots (every 40 ns) of CTCF bound to ssDNA on the C-rich (left) or G-rich strand (right) of the consensus motif sample by 1 μs MD simulations. The same visualization of CTCF bound to the *PDGFRA* G-rich strand can be found in Figure S19C. Corresponding pLDDT and PAE plots for the AlphaFold 3 initial conditions can be found in FigS19. **(G)** Simulated distribution of distance between center of mass of each ZF and its 3 nearest DNA bps for each CTCF ZF for the C-rich strand (left) or G-rich strand (right). N-terminal ZFs (1-6) are shown in blue and C-terminal ZFs (7-11) are shown in red. A second replicate of each is shown in Fig. S19G-H. **(H)** Left: experimental scheme of a TIRF assay to measure CTCF-JF646 binding on ssDNA and dsDNA. Right: representative images of CTCF-JF646 spots immobilized on ssDNA (top) (from left to right: G-rich strand, C-rich strand, non-specific) or dsDNA (bottom) (from left to right: consensus motif or non-specific) substrates. Additional representative images can be found in Figure S20A. **(I)** A quantification of the number of spots detected in **(H)**. Spot counts (mean ± s.e.m) are 735 ± 78 for consensus dsDNA, 246 ± 31 for control dsDNA, 620 ± 29 for the G-rich consensus strand, 225 ± 3 for the C-rich consensus strand, and 151 ± 12 for control ssDNA (n = 3 independent experiments for all conditions). **(J)** Left: experimental scheme of cyclical unzipping survival experiment where either high or low additional tension was applied after fully disrupting the CTCF-DNA complex. Right: representative cyclical unzipping experiments where CTCF dissociated after 2 cycles under high tension (black) and CTCF dissociated after 20 cycles under low tension (blue)**. (K)** A cumulative distribution curve depicting the number of cycles until CTCF dissociation for high- (black, median = 1, n = 25) and low-tension (blue, median = 15, n = 24).

### CTCF adopts a non-canonical, ssDNA-bound conformation to survive mechanical invasion

In addition to regulation by CpG methylation and nucleosome positioning, the CTCF-DNA complex must also contend with disruptive cellular processes at TAD boundaries, such as transcription, replication, and DNA repair, that transiently generate ssDNA. Uniquely, by creating an unzipping fork, the dynamic measurements we performed recapitulated these ssDNA intermediates (Fig. S3). During these experiments, we observed a fully-unwrapped, motif-bound state that recovered without full dissociation (Fig. S3C), suggesting a conformation not captured by available structures or MD simulations[20–22,25]. We therefore hypothesized that CTCF can adopt a highly dynamic, ssDNA-bound conformation with preserved motif tethering, but which differs from the canonical dsDNA state.

To test this hypothesis and characterize this state, we combined MD simulations, single-molecule total internal reflection fluorescence (TIRF) microscopy, and additional unzipping experiments.

All-atom MD simulations starting from AlphaFold 3[51] predictions of CTCF bound to either the G-rich or C-rich strand of its consensus motif (Fig. S1A) showed that ssDNA complexes were more mobile than dsDNA complexes (Fig. 1G,H, 2F,G, S2, S19). Notably, whereas the C-rich strand showed relatively uniform mobility across ZFs, the G-rich strand exhibited a dsDNA-like mobility profile (for both the consensus and *PDGFRA* motifs), with higher fluctuations at the termini and reduced mobility at the central ZFs. This result suggests that CTCF bound to the G-rich strand, but not the C-rich strand, may maintain a similar conformation as the CTCF-dsDNA complex, which could manifest as differences in CTCF's ability to bind the two strands.

To test this experimentally, we incubated fluorescently-labeled CTCF with different immobilized DNA substrates and visualized binding by single-molecule TIRF microscopy (Fig. 2H, S20A). Consistent with the simulations, C-rich strand binding resembled that of non-specific substrates, exhibiting minimal CTCF accumulation. In contrast, the G-rich strand supported ~80% as many binding events as the dsDNA motif (Fig. 2H,I, S20A-C), consistent with the preserved fluctuation profile of the G-rich strand in MD simulations and a recent observation of CTCF binding to non-canonical DNA structures[58].

To assess CTCF's resilience to mechanical perturbations applied during disruptive chromatin processes, we mechanically challenged CTCF-DNA complexes through cyclical unzipping and rezipping of DNA (Fig. S20D)[59,60]. After disrupting CTCF-DNA contacts, we applied variable amounts of additional tension to the ssDNA-bound CTCF complex. At high applied tension (25-30 pN), CTCF was rarely detected in subsequent cycles (1 cycle median survival) (Fig. 2J,K). In contrast, at low applied additional tension (10-15 pN), which is closer to the forces exerted by RNA polymerases[61,62], the median CTCF retention on ssDNA was 15 cycles (Fig. 2J,K). Importantly, upon recovery, CTCF-DNA contacts appear to reform in a similar way, with no significant change in rupture force as a function of cycles (Fig. S20E-G). These data indicate that the motif-tethered, ssDNA-bound state preserves sequence recognition and is mechanically persistent, potentially bookmarking[40] TAD boundaries and reducing the need for subsequent, slow 3D search during chromatin transactions.

### CTCF complexes adopt dynamic conformations in cells

Our MD simulations and single-molecule *in vitro* measurements suggested a mechanism in which CTCF's ZFs undergo spontaneous unwrapping and rewrapping. If ZFs are dynamically engaged with DNA in cells, the motif should be transiently accessible to other DNA-binding proteins[63,64]. To test this in a native chromatin context, we targeted Cas9 to multiple sites across the *PDGFRA* locus in permeabilized HEK293T cells (Fig. 3A, Materials and Methods). While static protein-DNA complexes, such as nucleosomes, have been shown to almost entirely ablate Cas9 cleavage[65,66] we observed at least 39% cleavage of DNA for each gRNA, suggesting that the site must remain accessible (Fig. 3A). The highest degree of cleavage was observed for gRNAs containing less overlap with the motif center, supporting our *in vitro* observation that peripheral ZFs unwrap more readily than the central ZFs.

To ask whether cleavage only occurred because of motif exposure from full dissociation of CTCF, we challenged the CTCF complex with catalytically-dead Cas9 (dCas9) in a well-controlled, single-molecule unzipping assay[67–69] (Fig. 3B-C). In this assay, distinct mechanical footprints enabled clear distinction between unbound DNA, CTCF-bound DNA, and dCas9-bound DNA[67] (Fig. 3B). Despite pre-bound CTCF occupying the same essential DNA interactions[70], dCas9 occupied the motif 62% of the time after one minute, indicating CTCF displacement (Fig. 3C). Within the same one-minute duration, unchallenged CTCF remained bound 79% of the time, suggesting its displacement would require dCas9 invasion exploiting spontaneous motif exposure within bound CTCF via ZF unwrapping. The ability of Cas9 to evict CTCF in cells and *in vitro* could have further implications for invasion by other DNA-binding proteins.

Motivated by Cas9's ability to invade CTCF complexes, we asked whether transient ZF unwrapping is a general property in cells. Leveraging the high GC-content of CTCF motifs, we performed GpC methylation footprinting coupled to single-molecule nanopore sequencing (NanoNOMe)[71,72] across >20,000 high-confidence human CTCF sites[73] (Fig. 3D, S21, Materials and Methods). Calculating the mean fraction of GpC-inaccessible reads across motifs revealed the characteristic CTCF footprint flanked by well-positioned nucleosomes[38,55,56,74], confirming that our assay probed protein-bound CTCF sites (Fig. 3E-F, S21B-C).

Inspecting single-molecule reads at single-ZF domain resolution, and removing unbound and nucleosomal reads[75], we classified molecules as either completely- (~60%) or partially-inaccessible (~40%) (Fig. 3E, S21D, Materials and Methods). On average, GpCs within central ZFs were less accessible than those at the periphery, consistent with frequent peripheral ZF unwrapping (Fig. 3F, S21). Conditional probability analysis at the single-molecule level revealed strong correlation between neighboring ZFs, particularly at the motif periphery (Fig. 3G), indicative of clustered ZF engagement. Notably, relative to unbound reads, nucleosomes were shifted away from CTCF, with a larger shift observed for static molecules than dynamic molecules (Fig. S21E). This suggests that, in addition to CTCF occupancy, CTCF conformation may influence local chromatin architecture. This conclusion is consistent with a recent preprint, which identified multiple CTCF-dependent chromatin states[76]. Given the large number of motifs and chromatin contexts examined, these results suggest that spontaneous ZF unwrapping and rewrapping is a general, functionally important property of cellular CTCF-DNA complexes.

**Fig. 3: CTCF complexes are intrinsically mobile in cells. (A)** A schematic representation of the Cas9-CTCF competition experiment in permeabilized HEK293T cells (left) and the resulting uncleaved fraction of DNA from different gRNAs targeting the *PDGFRA* motif (right). The gRNAs are depicted as red arrows pointing in the direction of R-loop propagation and the protospacer adjacent motifs (PAMs) as yellow circles. The size and location of the gRNAs (20 bp) and PAMs (3 bp) are to scale. The known locations of select ZFs are overlaid as gray bars. Data was normalized to Cas9 undigested control and is shown as mean, s.e.m +/- (n=2 for all gRNA). **(B)** Representative unzipping traces with cartoons of the *PDGFRA* motif unbound (black), CTCF-bound (left, red), or dCas9-bound (right, blue). Using the same gRNA 4 used in **(A)**, dCas9 was targeted to ZF4. **(C)** Left: schematic representation of the single-molecule optical tweezers competition experiment. Right: pie charts illustrating the percentage of measurements each outcome was observed: unbound DNA (gray), CTCF-bound DNA (red), or dCas9-bound DNA (blue). **(D)** Left: A schematic representation of the nanopore sequencing GpC footprinting assay in HEK293T cells. Right: The fraction of inaccessible DNA molecules averaged across 22,460 number of CTCF binding sites (+/- 200 bp). Grey shading indicates standard deviation across the sampled motifs (n=22,460) and blue shading indicates ZF footprint of the CTCF binding motif (Materials and Methods). **(E)** All single-molecule sequencing reads (n = 58) at a representative CTCF binding site (chr16:8792017-8792147). Black circles indicate unmethylated GpCs, white circles indicate methylated GpCs, and grey boxes indicate positions where methylation is not possible (no GpC) or uncalled. Reads are grouped, from top to bottom, as "inaccessible" (26%), “partially accessible” (17.2%) “nucleosomal” (42%)[75], or “unbound” (15%), percentages indicate across all sites (see Materials and Methods). Percentages shown as average from total reads sampled (n=1,0618,848). Excluding nucleosomal and unbound reads, inaccessible molecules make up about 60% of reads across all CTCF sites and partially accessible molecules make up the remaining about 40%. **(F)** Fraction unmethylated at each GpC within a CTCF site grouped by ZF triplet in nucleosomal and unbound excluded reads. Means ± standard deviation, with sample size as motifs(reads) for each ZF : ZF1 = 0.80 ± 0.25, n = 3,643(72,125); ZF2 = 0.85 ± 0.23, n = 1,020(16,999); ZF3 = 0.94 ± 0.13, n = 3,914(77,737); ZF4 = 0.96 ± 0.11, n = 9,147(184,500); ZF6 = 0.97 ± 0.08, n = 3,132(65,642); ZF7 = 0.96 ± 0.11, n = 3,916(85,908); ZF8 = 0.96 ± 0.09, n = 4,396(93,376); ZF9 = 0.90 ± 0.18, n = 1,905(36,131); ZF10 = 0.84 ± 0.23, n = 2,811(53,307); ZF11 = 0.78 ± 0.27, n = 2,837(53,615). The GpCs within the 20-60 bps proceeding (N-terminal nucleosome-depleted region (NDR)) or following (C-terminal NDR) CTCF sites are shown as negative controls to represent accessible chromatin. Mean +/- standard deviation are shown as error bars. The inset CTCF depiction is repeated from Fig. 1G MD simulations. ZF5 is excluded from the analysis due to low GpC coverage. **(G)** Conditional probability for a subset of partially accessible molecules of ZF j being bound (unmethylated GpC within the triplet) given that ZF i is bound (Materials and Methods). N(motifs): ZF1=2,229, ZF2=559, ZF3=2125, ZF4=6050, ZF6=2544, ZF7=2768, ZF8=2966, ZF9=984, ZF10=1457, ZF11=1265.

### PDS5A modulates CTCF conformations to reinforce TAD boundaries

Our *in vitro* and genomic data indicated that a partially-unwrapped form of CTCF, where central ZFs were engaged and peripheral ZFs were unbound, dominated the conformational landscape. If this state represents a cohesin-compatible conformation, cohesin-associated factors should be enriched at motifs that more frequently adopt this conformation. To test this hypothesis, we correlated GpC inaccessibility at each ZF with publicly available chromatin immunoprecipitation sequencing (ChIP-seq) datasets[77] for cohesin subunits[78].

Consistent with our hypothesis, occupancy of the STAG1, RAD21, and PDS5A cohesin subunits, as well as other expected chromatin features, were negatively correlated with peripheral ZF binding and positively correlated with central ZF binding[19,20] (Fig. 4A-B, Fig. S22). However, the ATPase-activating NIPBL subunit, although present, (Fig. S22H) did not exhibit this patterning and its depletion minimally affected RAD21-ZF correlations (Fig. 4A, Fig. S22A-B). Moreover, publicly available CTCF ChIP mass spectrometry (ChIP-MS) and ChIP selective isolation of chromatin-associated proteins (ChIP-SICAP) data[79,80] showed enrichment of PDS5A, but not NIPBL with chromatin-bound CTCF (Fig. 4C). Together, these results suggest that the partially-unwrapped CTCF conformation is predominately associated with PDS5A-cohesin on chromatin.

Because PDS5A-cohesin does not extrude DNA, yet PDS5A localizes to CTCF sites, we asked whether PDS5A engages motif-bound CTCF independently of cohesin. To examine PDS5A's cohesin-independent behavior, we employed a two-color optical-tweezers assay[81] to simultaneously track fluorescently-labeled CTCF and PDS5A-GFP on a long, taut DNA substrate harboring CTCF-binding sites (Fig. 4D, S23A). CTCF exhibited an initial, 1D sliding phase on DNA with a diffusion coefficient of 0.62 ± 0.45 $kb^2/s$, in line with a previous report[28] (Fig. S23B-D). Subsequently, CTCF's position stabilized, which we interpreted as binding to a motif (Fig. 4D, S23A-B). Once bound, CTCF residence time increased (18.5 s median for diffusive, 98.5 s median for bound) and was robust to 5-45 pN of tension (Fig. S23C-F). PDS5A also exhibited 1D sliding behavior on DNA with relatively quick dissociation (5.4 s median lifetime) (Fig. 4D-E, S23G). PDS5A was unable to bypass encountered diffusive or immobilized CTCF, either "bouncing" off or "docking" onto it (Fig. 4D, S23H-I). Notably, upon colocalization with immobilized CTCF, PDS5A residence time on DNA dramatically increased to a median lifetime of 67.8 s. Although we were blind to motif orientation, the presence of two discrete behaviors is consistent with the asymmetric barrier behavior of CTCF. Atomic force microscopy (AFM) imaging with CTCF and PDS5A also revealed enrichment of protein density at CTCF motif positions, suggesting direct association between two proteins on DNA (Fig. 4F, S24A-C). To better understand the structural basis of these interactions, we generated an AlphaFold 3 prediction of PDS5A in complex with *PDGFRA*-bound CTCF. In addition to recapitulating the known interaction at CTCF's N-terminal IDR[30], the model predicted additional, albeit low confidence, contacts between PDS5A and CTCF's ZFs (Fig. 4G, S24F-K), raising the possibility of direct ZF modulation by PDS5A.

Indeed, PDS5A shifted the conformational ensemble of CTCF towards the partially-unwrapped conformation (from 76% to 83% occupancy) to levels comparable to the consensus sequence (82% occupancy) (Fig. 2A, 4H). This enrichment of the productive conformation may serve as a "priming" mechanism to facilitate cohesin capture, but remains to be demonstrated during active extrusion. Furthermore, in our single-molecule unzipping assay, PDS5A mechanically stabilized CTCF-DNA complexes (Fig. 4I-L, S25). When probed from the C-terminal orientation, PDS5A increased CTCF's rupture force, which was abolished upon N-terminal IDR truncation, consistent with its established role in PDS5A recruitment (Fig. 4K)[30]. In addition, in the presence of PDS5A, we observed a distinct population of CTCF-DNA complexes exhibiting an exceptionally high N-terminal rupture force (mean = 35.2 ± 3.2 pN) (Fig. 4J cyan points, 4L, S25B), far above the 15–25 pN range reported for other TFs[47,53,59,82]. Consistently, in dynamic measurements, PDS5A caused CTCF to transiently explore a more tightly bound state (1.2% occupancy) (Fig. S25N-O).

The inclusion of a cohesin complex (SMC1a, SMC3, RAD21, and STAG1) increased the abundance of the high rupture force population from 3.1% to 12.0% and the tightly-bound conformation from 1.2% to 5.6% occupancy (Fig. 4I-L, S25C, S25N-Q). No effect was observed on the mechanical strength or conformational dynamics of CTCF with cohesin alone. Furthermore, NIPBL (with verified extrusion capability (Fig. S24E-F)) ablated PDS5A-mediated mechanical stabilization of CTCF ZFs, consistent with direct competition between the two factors (Fig. 4J, S25A-J) as observed in a recent preprint.[83] These results suggest that PDS5A modulates CTCF ZFs, possibly to facilitate cohesin capture and stabilize the complex at loop anchors. Indeed, comparison of available Hi-C data before and after PDS5A depletion at the constitutive *PDGFRA* insulator[84] showed a reduction in CTCF-mediated insulation (Fig. 4M), further supporting the proposed role of PDS5A in cohesin-anchoring at CTCF[12,48,83,84]. Together, these findings establish PDS5A as a direct regulator of CTCF ZF-DNA interaction strength and conformational dynamics, possibly enhancing CTCF's capacity to recruit cohesin.

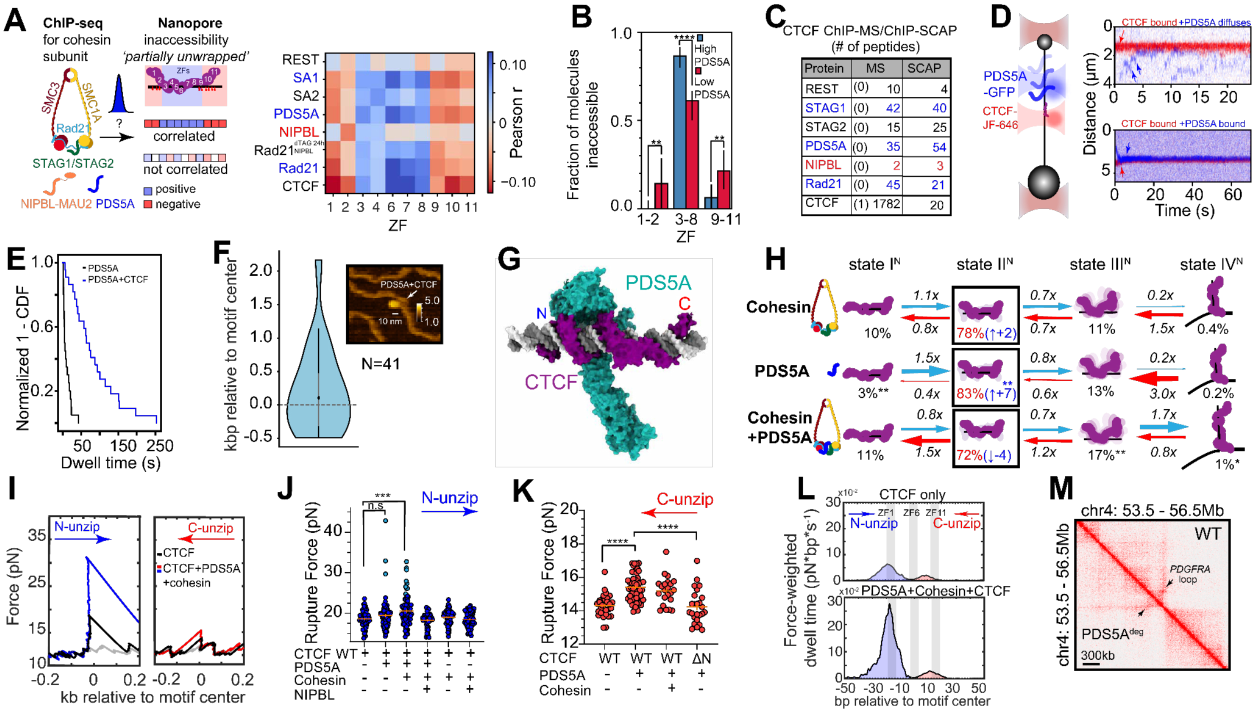

**Fig: 4: The cohesin subunit PDS5A modulates CTCF conformations to reinforce TAD boundaries.** **(A)** Left: cartoon of cohesin subunits. Schematic representation of the ChIP-seq and nanopore footprinting correlation analysis. Right: Above, CTCF depiction is repeated from Fig. 1G MD simulations. Below,

Pearson correlation between publicly available ChIP-seq data sets of cohesin subunits in retinal pigment epithelial cells (RPE1) cells from Popay et al[77] and REST (HEK293), as a negative control (downloaded from the ENCODE portal www.encodeproject.org with the identifier ENCFF981ILL), and GpC inaccessibility for a subset of partially-accessible molecules (as shown in Fig. 3D-G) at each ZF. See Fig. S22 for additional proteins, cell types, and significance levels. **(B)** GpC inaccessibility within ZF clusters (1-2, 3-8, 9-11) for CTCF sites with the least (1st decile "Low PDS5A", red) and most (10th decile, "High PDS5A", blue) PDS5A ChIP signal. Data is shown as median (95% confidence intervals (CI)). Low PDS5A (ZF1-2, ZF3-8, ZF9-11): 0.14 (0-0.29 CI) (n=207), 0.61 (0.5-0.67 CI) (n=616), 0.21 (0.1-0.33 CI) (n=363). High PDS5A (ZF1-2, ZF3-8, ZF9-11): 0 (0–0.05 CI) (n=298, p = 0.0013), 0.87 (0.8-0.92 CI) (n=687, p<0.0001), 0.06 (0-0.136 CI) (n=399, p = 0.0068). P-values are calculated with a two-tailed Mann-Whitney test, with significance indicated as **p<0.01, ****p<0.0001. **(C)** Table of CTCF ChIP-MS and ChIP-SCAP values of cohesin subunits in mouse embryonic stem cells obtained from Ortabozkoyun et al [79] and from Dehingia et al[80]. **(D)** Left: schematic representation of an optical tweezers assay where CTCF-JF646 and PDS5A-GFP binding and sliding are imaged with confocal scanning on long, taut dsDNA. Right: representative kymographs of PDS5A encountering stably-bound CTCF binding. The top kymograph shows transient PDS5A interaction (or "bouncing"), while the bottom kymograph shows continuous colocalization for more than 60 seconds (or "docking"). **(E)** 1-CDF of dwell times of PDS5A without CTCF (black; median = 5.4s, n = 20) and colocalized with CTCF (red; median = 67.8s, n = 22). **(F)** Binding position of PDS5A, as determined by AFM, on a symmetric DNA substrate containing 2 CTCF consensus motifs (median = 106 bp, -160 bp to 297 bp 95% CI, n = 41). Position is given as distance from the nearest CTCF binding site. The inset displays a representative AFM image of PDS5A colocalized with DNA at the expected CTCF binding motif (see Materials and Methods). **(G)** AlphaFold 3 predicted structure of CTCF (purple) bound to *PDGFRA* dsDNA (grey) and PDS5A (turquoise). For visualization purposes, both IDRs are hidden. Corresponding pLDDT and PAE plots can be found in FigS24. **(H)** Transition rates between four confirmation states of CTCF in the presence of cohesin (top), PDS5A (middle) and cohesin + PDS5A (bottom). Black boxes and red text indicate occupancy of the most occupied state. Other occupancy percentages are written in black. All transition rates and occupancies can be found in Figure S12, Figure S14, and Table S2. P-values are calculated with a two-tailed Mann-Whitney test relative to N-terminal WT CTCF bound to *PDGFRA* and can be found in Table S2, with significance indicated as *p<0.05, **p<0.01. **(I)** Representative unzipping traces of CTCF in the presence of PDS5A and cohesin from the N-terminal (left, blue) and C-terminal (right, red) orientation. For comparison, representative unbound DNA (gray) and CTCF-only (black) unzipping traces are shown. **(J)** Rupture forces of N-terminal unzipping of WT CTCF in the presence and absence of PDS5A, cohesin, and NIPBL. Mean rupture forces are 18.6 ± 0.3 pN (n=57) for WT CTCF (as shown in Fig. 1E), 19.4 ± 0.4 pN (n=96) for WT CTCF and PDS5A, 20.5 ± 0.4 pN (n=83) for WT CTCF, PDS5A, and cohesin, 18.2 ± 0.3 pN (n=46) for WT CTCF, PDS5A, NIPBL, and cohesin, 19.0 ± 0.2 pN (n=76) for WT CTCF and cohesin, 18.5 ± 0.2 pN (n=66) for WT CTCF, NIPBL, and cohesin. Data are mean ± standard error. Points shown in cyan were predicted to be within a higher rupture force population (Materials and Methods). For WT CTCF and PDS5A, populations had mean rupture forces of 18.9 ± 0.2 pN (n=93) and 35.2 ± 3.2 pN (n=3). For WT CTCF, PDS5A, and cohesin populations had mean rupture forces of 19.6 ± 0.3 pN (n=73) and 27.8 ± 0.9 pN (n=10). **(K)** Rupture forces of C-terminal unzipping of WT and ΔN CTCF in the presence and absence of PDS5A and cohesin. Mean rupture forces are 14.3 ± 0.1 pN (n=31) for WT CTCF (as shown in Fig. 1E), 15.5 ± 0.1 pN (n=52) for WT CTCF and PDS5A, 15.2 ± 0.2 pN (n=22) for WT CTCF, PDS5A, and cohesin, 14.2 ± 0.2 pN (n=25) for ΔN CTCF and PDS5A. Data are mean ± standard error. Using a 1-D, two-state K-S test, significance is indicated as follows: ***p<0.001, ****p<0.0001 **(L)** Force-weighted dwell time maps of CTCF bound to the *PDGFRA* motif (red) and the CTCF-PDS5A-cohesin complex (blue). For the N-terminal orientation of the CTCF-PDS5A-cohesin complex, the higher rupture force population denoted in **(J)** is depicted. The locations of ZFs are overlaid as gray bars. **(M)** Hi-C map of the *PDGFRA* locus in PLC/PRF/5 cells with and without induced PDS5A FKBP12F36V-dTAG degron. Data was obtained from Yu et al[84].

### DISCUSSION

We propose that CTCF is not simply a static insulating factor, but serves as a tunable, semi-permeable barrier that samples productive and unproductive conformations on timescales comparable to the duration of CTCF's encounter with extruding cohesin (Fig. 5). Although remaining to be directly tested, we suggest that this conformational ensemble integrates cues from the chromatin environment to regulate cohesin stalling by transiently masking or exposing a binding interface needed for productive cohesin capture.

Cohesin is present in two mutually exclusive forms[78]: an extruding NIPBL-cohesin complex and a non-extruding PDS5-cohesin complex. Consistent with a previously hypothesized two-step mechanism for cohesin capture[43], we propose that free PDS5 first associates with CTCF, "priming" it for orientation-dependent cohesin engagement. Subsequently, CTCF-bound PDS5 accelerates NIPBL dissociation[83], converting cohesin from its extruding form to a mechanically-stabilized form, potentially reinforcing loop boundaries[12,43,83].

Requiring a fine balance between stability and flexibility, CTCF enables cells to maintain long-lived loops, while remaining adaptable to an ever-changing chromatin environment. The local bp-scale conformational exploration of DNA-anchored CTCF, modulated by chromatin context and cofactors, establishes a kinetic principle[85] for how CTCF establishes, maintains, and modulates chromatin organization and function of the genome. The generalizability of this conformationally-tuned "local search" principle to the regulation of cofactor recruitment to other sequence-specific TFs warrants further investigation.

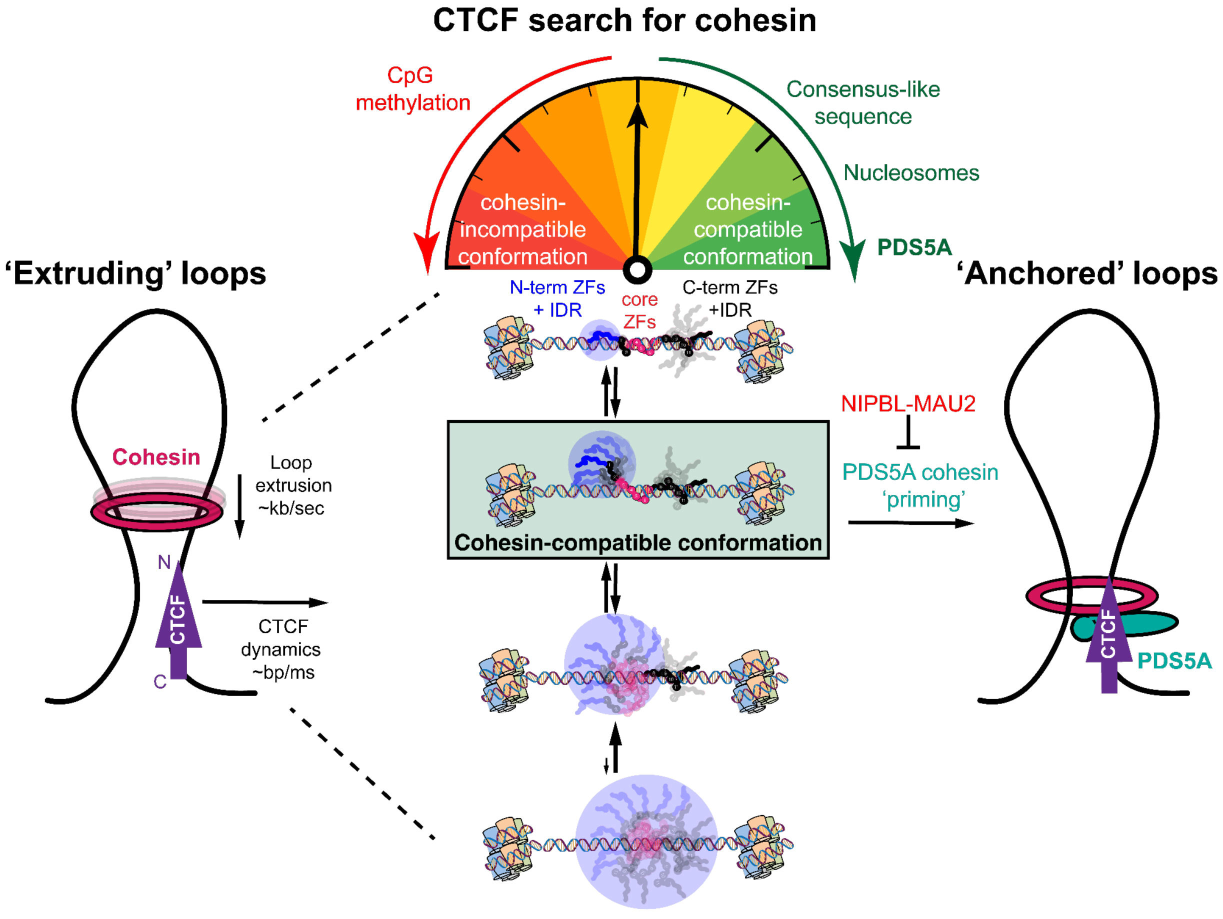

**Figure 5. CTCF conformational dynamics regulate TAD boundaries.** CTCF samples product and unproductive conformations on the millisecond timescale through sequential unwrapping and rewrapping of blocks of ZFs. These conformations provide a framework for how CTCF balances stability and flexibility to regulate genome folding. This allows CTCF to adopt a ssDNA conformation, which enables rapid recovery of loop anchors without a new 3D search during disruptive processes. The chromatin environment shifts this conformational landscape, with CpG methylation increasing cohesin-incompatible conformations and weakening insulation, while consensus-like sequences, nucleosomes, and the cohesin subunit PDS5A stabilizing a cohesin-compatible conformation. In addition to priming CTCF for cohesin search, PDS5A stabilizes CTCF-cohesin complexes, forming highly-stabilized loop boundaries.

### ACKNOWLEDGMENTS

The authors thank Mathias Madalinski for HPLC purification of the JF646 HaloTag ligand; Andrew Drabeck for STAG1 purification and msfGFP-PDS5A cloning; Maciej Zakzec for cloning of N- and C-terminal CTCF deletion constructs; and Anders Hansen and Gina M. Dailey (from the laboratories of Robert Tjian and Xavier Darzacq) for providing a plasmid for mouse CTCF expression. We are grateful to the laboratories of Sua Myong, Bradley Bernstein, Anders Hansen, Frederick Alt, and Carl Wu for critical discussions. We thank Cees Dekker, Hylkje Geertsema, and Jos Zwanikken for their comments during T.J.R.K.'s thesis defense, and Brian Analikwu for advice during his thesis. We also thank Xiaona Tang and colleagues in the Carl Wu laboratory for server access, and Roman Barth for technical advice on in vitro loop-extrusion assays. We acknowledge the laboratory of Jesse Dixon for generating cohesin-subunit ChIP-seq data. We thank David Mohr and the Genetic Resources Core Facility (GRCF) at Johns Hopkins University for performing NextSeq and NovaSeq runs. We thank Yana Li of the Johns Hopkins Eukaryotic Tissue Culture Facility for expressing mouse CTCF. Finally, we thank the Hatzakis, Farnung, Timp, Aksimentiev, Peters, Vos, and Ha laboratories for stimulating discussions throughout the project. J-MP is also an adjunct professor at the Medical University of Vienna and an external member of the Max Planck Institute of Biochemistry, Martinsried. This article is subject to HHMI's Immediate Access to Research policy, which requires that this article be made publicly available as initial and revised preprints deposited on a designated preprint server under a CC BY 4.0 license.

### FUNDING

This work was supported by a European Molecular Biology Organization (EMBO) fellowship (ALTF 825-2021) and a Human Frontier Science Program (HFSP) fellowship (LT0049/2022-L) to S.R. H.C. acknowledges support from the Beckman Fellowship, UIUC. H.C. and A.A. acknowledge support for supercomputer time provided through TACC Frontera (MCB20012) and ACCESS allocation grant (MCA05S028). A.A. and T.H. acknowledge support from the National Science Foundation Science and Technology Center for Quantitative Cell Biology (NSF STC-QCB, Grant No. 2243257). N.S.H. acknowledges funding from the Novo Nordisk foundation challenge center for Optimised Oligo escape (NNF23OC0081287), Novo Nordisk foundation Center for 4D cellular dynamics (NNF22OC0075851) and Villum foundation experiment (40801). T.H. received support from the National Institutes of Health (R35 GM122569, U01 DK127432). T.H., L.F. and S.M.V. are employees of the Howard Hughes Medical Institute. Research in the laboratory of J.-M.P. is supported by Boehringer Ingelheim, the European Union (ERC AdG 101020558 LoopMechRegFun, MSCA 101072505 CohesiNet) and the Vienna Science and Technology Fund (LS19-029).

### AUTHOR CONTRIBUTIONS

S.R., P.J.M., E.W.S. and T.J.R.K. contributed equally to this work. S.R., P.J.M. and T.H. conceived the project. S.R., P.J.M. and T.J.R.K. generated molecular constructs and performed optical tweezers measurements, and S.R. and P.J.M. analyzed rupture-force data. E.W.S. performed analysis of dynamic measurements and quantified AFM data. H.C. and A.A. performed and analyzed MD simulations. I.F.D. expressed, purified and labelled human CTCF and mutants, and J.S.Z. purified mouse CTCF. L.C. expressed and purified cohesin and PDS5A, and M.O.-V. expressed and purified NIPBL. P.M. and J.H. reconstituted nucleosomal constructs. R.M.-U., P.M., P.J.M. and S.R. performed single-molecule TIRF experiments. S.P., P.J.M. and S.R. performed confocal optical tweezers and AFM measurements. P.J.M. performed AlphaFold3 structure predictions. S.R. performed Cas9 accessibility assays in cells. S.R. and P.W.H. performed nanopore sequencing, and S.R. analyzed the nanopore data with input from P.J.M., E.W.S. and P.W.H. S.R. carried out and analyzed CUT&RUN and MNase–seq, and mined ChIP–seq, ChIP–MS, ChIP–SICAP and Hi–C datasets. N.S.H., W.T., L.F., S.M.V., J.-M.P. and A.A. provided discussion, expertise and support for collaborations. S.R., P.J.M. and E.W.S. wrote the manuscript in coordination with T.H. and with input from J.-M.P. and I.F.D. T.H. supervised the project. All authors discussed the results and commented on the manuscript.

### COMPETING INTERESTS

Authors declare that they have no competing interests.

## References

1. Nora, E. P. *et al.* Spatial partitioning of the regulatory landscape of the X-inactivation centre. *Nature* **485**, 381–385 (2012).

2. Szabo, Q., Bantignies, F. & Cavalli, G. Principles of genome folding into topologically associating domains. *Sci. Adv.* **5**, eaaw1668 (2019).

3. Rao, S. S. P. *et al.* A 3D Map of the Human Genome at Kilobase Resolution Reveals Principles of Chromatin Looping. *Cell* **159**, 1665–1680 (2014).

4. Dixon, J. R. *et al.* Topological domains in mammalian genomes identified by analysis of chromatin interactions. *Nature* **485**, 376–380 (2012).

5. Parelho, V. *et al.* Cohesins functionally associate with CTCF on mammalian chromosome arms. *Cell* **132**, 422–433 (2008).

6. Wendt, K. S. *et al.* Cohesin mediates transcriptional insulation by CCCTC-binding factor. *Nature* **451**, 796–801 (2008).

7. Busslinger, G. A. *et al.* Cohesin is positioned in mammalian genomes by transcription, CTCF and Wapl. *Nature* **544**, 503–507 (2017).

8. de Wit, E. *et al.* CTCF Binding Polarity Determines Chromatin Looping. *Mol. Cell* **60**, 676–684 (2015).

9. Sanborn, A. L. *et al.* Chromatin extrusion explains key features of loop and domain formation in wild-type and engineered genomes. *Proc. Natl. Acad. Sci.* **112**, E6456–E6465 (2015).

10. Davidson, I. F. *et al.* DNA loop extrusion by human cohesin. *Science* **366**, 1338–1345 (2019).

11. Kim, Y., Shi, Z., Zhang, H., Finkelstein, I. J. & Yu, H. Human cohesin compacts DNA by loop extrusion. *Science* **366**, 1345–1349 (2019).

12. Wutz, G. *et al.* Topologically associating domains and chromatin loops depend on cohesin and are regulated by CTCF, WAPL, and PDS5 proteins. *EMBO J.* **36**, 3573–3599 (2017).

13. Filippova, G. N. *et al.* An Exceptionally Conserved Transcriptional Repressor, CTCF, Employs Different Combinations of Zinc Fingers To Bind Diverged Promoter Sequences of Avian and Mammalian c-myc Oncogenes. *Mol. Cell. Biol.* **16**, 2802–2813 (1996).

14. Lupiáñez, D. G. *et al.* Disruptions of Topological Chromatin Domains Cause Pathogenic Rewiring of Gene-Enhancer Interactions. *Cell* **161**, 1012–1025 (2015).

15. Solé-Ferran, M. & Losada, A. Cohesin in 3D: development, differentiation, and disease. *Genes Dev.* **39**, 679–696 (2025).

16. Flavahan, W. A. *et al.* Insulator dysfunction and oncogene activation in IDH mutant gliomas. *Nature* **529**, 110–114 (2016).

17. Monteagudo-Sánchez, A., Noordermeer, D. & Greenberg, M. V. C. The impact of DNA methylation on CTCF-mediated 3D genome organization. *Nat. Struct. Mol. Biol.* **31**, 404–412 (2024).

18. Lobanenkov, V. V. *et al.* A novel sequence-specific DNA binding protein which interacts with three regularly spaced direct repeats of the CCCTC-motif in the 5'-flanking sequence of the chicken c-myc gene. *Oncogene* **5**, 1743–1753 (1990).

19. Hashimoto, H. *et al.* Structural Basis for the Versatile and Methylation-Dependent Binding of CTCF to DNA. *Mol. Cell* **66**, 711-720.e3 (2017).

20. Yang, J. *et al.* Structures of CTCF–DNA complexes including all 11 zinc fingers. *Nucleic Acids Res.* **51**, 8447–8462 (2023).

21. Yin, M. *et al.* Molecular mechanism of directional CTCF recognition of a diverse range of genomic sites. *Cell Res.* **27**, 1365–1377 (2017).

22. Zhang, H. *et al.* CTCF and R-loops are boundaries of cohesin-mediated DNA looping. *Mol. Cell* **83**, 2856-2871.e8 (2023).

23. Nora, E. P. *et al.* Targeted Degradation of CTCF Decouples Local Insulation of Chromosome Domains from Genomic Compartmentalization. *Cell* **169**, 930-944.e22 (2017).

24. Hansen, A. S., Pustova, I., Cattoglio, C., Tjian, R. & Darzacq, X. CTCF and cohesin regulate chromatin loop stability with distinct dynamics. *eLife* **6**, e25776 (2017).

25. Nakahashi, H. *et al.* A Genome-wide Map of CTCF Multivalency Redefines the CTCF Code. *Cell Rep.* **3**, 1678–1689 (2013).

26. Rauluseviciute, I. *et al.* JASPAR 2024: 20th anniversary of the open-access database of transcription factor binding profiles. *Nucleic Acids Res.* **52**, D174–D182 (2024).

27. Martinez, S. R. & Miranda, J. L. CTCF terminal segments are unstructured. *Protein Sci.* **19**, 1110–1116 (2010).

28. Davidson, I. F. *et al.* CTCF is a DNA-tension-dependent barrier to cohesin-mediated loop extrusion. *Nature* **616**, 822–827 (2023).

29. Li, Y. *et al.* The structural basis for cohesin–CTCF-anchored loops. *Nature* **578**, 472–476 (2020).

30. Nora, E. P. *et al.* Molecular basis of CTCF binding polarity in genome folding. *Nat. Commun.* **11**, 5612 (2020).

31. Nishana, M. *et al.* Defining the relative and combined contribution of CTCF and CTCFL to genomic regulation. *Genome Biol.* **21**, 108 (2020).

32. Pugacheva, E. M. *et al.* CTCF mediates chromatin looping via N-terminal domain-dependent cohesin retention. *Proc. Natl. Acad. Sci.* **117**, 2020–2031 (2020).

33. Hansen, A. S., Cattoglio, C., Darzacq, X. & Tjian, R. Recent evidence that TADs and chromatin loops are dynamic structures. *Nucleus* **9**, 20–32 (2018).

34. Petela, N. J. *et al.* Scc2 Is a Potent Activator of Cohesin's ATPase that Promotes Loading by Binding Scc1 without Pds5. *Mol. Cell* **70**, 1134-1148.e7 (2018).

35. Kikuchi, S., Borek, D. M., Otwinowski, Z., Tomchick, D. R. & Yu, H. Crystal structure of the cohesin loader Scc2 and insight into cohesinopathy. *Proc. Natl. Acad. Sci.* **113**, 12444–12449 (2016).

36. Barth, R. *et al.* Two CTCF motifs impede cohesin-mediated DNA loop extrusion. 2025.01.26.634934 Preprint at https://doi.org/10.1101/2025.01.26.634934 (2025).

37. Do, C. & Skok, J. A. Factors that determine cell type–specific CTCF binding in health and disease. *Curr. Opin. Genet. Dev.* **88**, 102244 (2024).

38. Tajmul, M. *et al.* CTCF binding landscape is shaped by the epigenetic state of the N-terminal nucleosome in relation to CTCF motif orientation. *Nucleic Acids Res.* **53**, gkaf587 (2025).

39. Shukla, S. *et al.* CTCF-promoted RNA polymerase II pausing links DNA methylation to splicing. *Nature* **479**, 74–79 (2011).

40. Owens, N. *et al.* CTCF confers local nucleosome resiliency after DNA replication and during mitosis. *eLife* **8**, e47898 (2019).

41. Hilmi, K. *et al.* CTCF facilitates DNA double-strand break repair by enhancing homologous recombination repair. *Sci. Adv.* **3**, e1601898 (2017).

42. Lee, J. *et al.* Kinetic organization of the genome revealed by ultra-resolution, multiscale live imaging. 2025.03.27.645817 Preprint at https://doi.org/10.1101/2025.03.27.645817 (2025).

43. Hansen, A. S. CTCF as a boundary factor for cohesin-mediated loop extrusion: evidence for a multi-step mechanism. *Nucleus* **11**, 132–148 (2020).

44. Berghuis, B. A. *et al.* Strand separation establishes a sustained lock at the Tus–Ter replication fork barrier. *Nat. Chem. Biol.* **11**, 579–585 (2015).

45. Koch, S. J., Shundrovsky, A., Jantzen, B. C. & Wang, M. D. Probing protein-DNA interactions by unzipping a single DNA double helix. *Biophys. J.* **83**, 1098–1105 (2002).

46. Hall, M. A. *et al.* High-resolution dynamic mapping of histone-DNA interactions in a nucleosome. *Nat. Struct. Mol. Biol.* **16**, 124–129 (2009).

47. Rudnizky, S. *et al.* Single-molecule DNA unzipping reveals asymmetric modulation of a transcription factor by its binding site sequence and context. *Nucleic Acids Res.* **46**, 1513–1524 (2018).

48. Wutz, G. *et al.* ESCO1 and CTCF enable formation of long chromatin loops by protecting cohesinSTAG1 from WAPL. *eLife* **9**, e52091 (2020).

49. Marin-Gonzalez, A. *et al.* Cohesin drives chromatin scanning during the RAD51-mediated homology search. 2025.02.10.637451 Preprint at https://doi.org/10.1101/2025.02.10.637451 (2025).

50. Stella, S. *et al.* Conformational Activation Promotes CRISPR-Cas12a Catalysis and Resetting of the Endonuclease Activity. *Cell* **175**, 1856-1871.e21 (2018).

51. Abramson, J. *et al.* Accurate structure prediction of biomolecular interactions with AlphaFold 3. *Nature* **630**, 493–500 (2024).

52. Khamis, H., Rudnizky, S., Melamed, P. & Kaplan, A. Single molecule characterization of the binding kinetics of a transcription factor and its modulation by DNA sequence and methylation. *Nucleic Acids Res.* **49**, 10975–10987 (2021).

53. Strugo, N. *et al.* Intrinsically disordered regions facilitate Msn2 target search to drive promoter selectivity. 2025.05.23.654710 Preprint at https://doi.org/10.1101/2025.05.23.654710 (2025).

54. Khoury, A. *et al.* Constitutively bound CTCF sites maintain 3D chromatin architecture and long-range epigenetically regulated domains. *Nat. Commun.* **11**, 54 (2020).

55. Clarkson, C. T. *et al.* CTCF-dependent chromatin boundaries formed by asymmetric nucleosome arrays with decreased linker length. *Nucleic Acids Res.* **47**, 11181–11196 (2019).

56. Wiechens, N. *et al.* The Chromatin Remodelling Enzymes SNF2H and SNF2L Position Nucleosomes adjacent to CTCF and Other Transcription Factors. *PLoS Genet.* **12**, e1005940 (2016).

57. Lowary, P. T. & Widom, J. New DNA sequence rules for high affinity binding to histone octamer and sequence-directed nucleosome positioning. *J. Mol. Biol.* **276**, 19–42 (1998).

58. Samaniego-Castruita, D. *et al.* CTCF directly binds G-quadruplex structures to regulate genome topology and gene expression. 2025.02.03.636329 Preprint at https://doi.org/10.1101/2025.02.03.636329 (2025).

59. Rudnizky, S., Khamis, H., Malik, O., Melamed, P. & Kaplan, A. The base pair-scale diffusion of nucleosomes modulates binding of transcription factors. *Proc. Natl. Acad. Sci.* **116**, 12161–12166 (2019).

60. Rudnizky, S. *et al.* Extended and dynamic linker histone-DNA Interactions control chromatosome compaction. *Mol. Cell* **81**, 3410-3421.e4 (2021).

61. Nago, N., Rudnizky, S. & Kaplan, A. Asymmetric modulation of RNA polymerase elongation by a transcription factor. *Biophys. J.* **123**, 166a (2024).

62. Le, T. T. *et al.* Mfd Dynamically Regulates Transcription via a Release and Catch-Up Mechanism. *Cell* **172**, 344-357.e15 (2018).

63. Li, G. & Widom, J. Nucleosomes facilitate their own invasion. *Nat. Struct. Mol. Biol.* **11**, 763–769 (2004).

64. Polach, K. J. & Widom, J. Mechanism of protein access to specific DNA sequences in chromatin: a dynamic equilibrium model for gene regulation. *J. Mol. Biol.* **254**, 130–149 (1995).

65. Horlbeck, M. A. *et al.* Nucleosomes impede Cas9 access to DNA in vivo and in vitro. *eLife* **5**, e12677.

66. Yarrington, R. M., Verma, S., Schwartz, S., Trautman, J. K. & Carroll, D. Nucleosomes inhibit target cleavage by CRISPR-Cas9 in vivo. *Proc. Natl. Acad. Sci.* **115**, 9351–9358 (2018).

67. Zhang, Q. *et al.* The post-PAM interaction of RNA-guided spCas9 with DNA dictates its target binding and dissociation. *Sci. Adv.* **5**, eaaw9807 (2019).

68. Hall, P. M. *et al.* Polarity of the CRISPR roadblock to transcription. *Nat. Struct. Mol. Biol.* **29**, 1217–1227 (2022).

69. Rudnizky, S. *et al.* H2A.Z controls the stability and mobility of nucleosomes to regulate expression of the LH genes. *Nat. Commun.* **7**, 12958 (2016).

70. Jinek, M. *et al.* Structures of Cas9 Endonucleases Reveal RNA-Mediated Conformational Activation. *Science* **343**, 1247997 (2014).

71. Lee, I. *et al.* Simultaneous profiling of chromatin accessibility and methylation on human cell lines with nanopore sequencing. *Nat. Methods* **17**, 1191–1199 (2020).

72. Hook, P. W. & Timp, W. Beyond assembly: the increasing flexibility of single-molecule sequencing technology. *Nat. Rev. Genet.* **24**, 627–641 (2023).

73. Grant, C. E., Bailey, T. L. & Noble, W. S. FIMO: scanning for occurrences of a given motif. *Bioinformatics* **27**, 1017–1018 (2011).

74. Fu, Y., Sinha, M., Peterson, C. L. & Weng, Z. The insulator binding protein CTCF positions 20 nucleosomes around its binding sites across the human genome. *PLoS Genet.* **4**, e1000138 (2008).

75. Sönmezer, C. *et al.* Molecular Co-occupancy Identifies Transcription Factor Binding Cooperativity In Vivo. *Mol. Cell* **81**, 255-267.e6 (2021).

76. Do, C. *et al.* The coordination between CTCF, cohesin and TFs impacts nucleosome repositioning and chromatin insulation to define state specific 3D chromatin folding. 2024.11.02.620823 Preprint at https://doi.org/10.1101/2024.11.02.620823 (2025).

77. Popay, T. M. *et al.* Genome-wide in vivo dynamics of cohesin-mediated loop extrusion and its role in transcription activation. 2024.10.02.616323 Preprint at https://doi.org/10.1101/2024.10.02.616323 (2024).

78. Davidson, I. F. & Peters, J.-M. Genome folding through loop extrusion by SMC complexes. *Nat. Rev. Mol. Cell Biol.* **22**, 445–464 (2021).

79. Ortabozkoyun, H. *et al.* CRISPR and biochemical screens identify MAZ as a cofactor in CTCF-mediated insulation at Hox clusters. *Nat. Genet.* **54**, 202–212 (2022).

80. Dehingia, B. *et al.* RNA-binding proteins mediate the maturation of chromatin topology during differentiation. *Nat. Cell Biol.* **27**, 1510–1525 (2025).

81. Carcamo, C. C. *et al.* ATP binding facilitates target search of SWR1 chromatin remodeler by promoting one-dimensional diffusion on DNA. *eLife* **11**, e77352 (2022).

82. Li, M. *et al.* Dynamic regulation of transcription factors by nucleosome remodeling. *eLife* **4**, e06249 (2015).

83. Wutz, G. *et al.* PDS5 proteins control genome architecture by limiting the lifetime of cohesin-NIPBL complexes. 2025.08.30.673243 Preprint at https://doi.org/10.1101/2025.08.30.673243 (2025).

84. Yu, D. *et al.* Regulation of cohesin-mediated chromosome folding by PDS5 in mammals. *EMBO Rep.* **23**, e54853 (2022).

85. Rudnizky, S., Murray, P. J., Wolfe, C. H. & Ha, T. Single-Macromolecule Studies of Eukaryotic Genomic Maintenance. *Annu. Rev. Phys. Chem.* **75**, 209–230 (2024).

86. Weissmann, F. *et al.* biGBac enables rapid gene assembly for the expression of large multisubunit protein complexes. *Proc. Natl. Acad. Sci. U. S. A.* **113**, E2564-2569 (2016).

87. Gibson, D. G. *et al.* Enzymatic assembly of DNA molecules up to several hundred kilobases. *Nat. Methods* **6**, 343–345 (2009).

88. Weissmann, F. & Peters, J.-M. Expressing Multi-subunit Complexes Using biGBac. in *Protein Complex Assembly: Methods and Protocols* (ed. Marsh, J. A.) 329–343 (Springer, New York, NY, 2018). doi:10.1007/978-1-4939-7759-8_21.

89. Vos, S. M. *et al.* Architecture and RNA binding of the human negative elongation factor. *eLife* **5**, e14981 (2016).

90. Hansen, A. S., Amitai, A., Cattoglio, C., Tjian, R. & Darzacq, X. Guided nuclear exploration increases CTCF target search efficiency. *Nat. Chem. Biol.* **16**, 257–266 (2020).

91. Gradia, S. *et al.* MacroBac: New technologies for robust and efficient large-scale production of recombinant multi-protein complexes. *Methods Enzymol.* **592**, 1–26 (2017).

92. Farnung, L., Vos, S. M., Wigge, C. & Cramer, P. Nucleosome–Chd1 structure and implications for chromatin remodelling. *Nature* **550**, 539–542 (2017).

93. Poyton, M. F. *et al.* Coordinated DNA and histone dynamics drive accurate histone H2A.Z exchange. *Sci. Adv.* **8**, eabj5509 (2022).

94. Paul, T. & Myong, S. Protocol for generation and regeneration of PEG-passivated slides for single-molecule measurements. *STAR Protoc.* **3**, 101152 (2022).

95. CUTANA$^{TM}$ CUT&RUN Protocol | Watch CUT&RUN Protocol Walk-Through. https://www.epicypher.com/resources/protocols/cutana-cut-and-run-protocol/.

96. Ewels, P. A. *et al.* The nf-core framework for community-curated bioinformatics pipelines. *Nat. Biotechnol.* **38**, 276–278 (2020).

97. nf-core/mnaseseq. nf-core (2023).

98. Chen, K. *et al.* DANPOS: Dynamic analysis of nucleosome position and occupancy by sequencing. *Genome Res.* **23**, 341–351 (2013).

99. Simpson, J. T. *et al.* Detecting DNA cytosine methylation using nanopore sequencing. *Nat. Methods* **14**, 407–410 (2017).

100. Dekker, J. *et al.* The 4D nucleome project. *Nature* **549**, 219–226 (2017).

101. Reiff, S. B. *et al.* The 4D Nucleome Data Portal as a resource for searching and visualizing curated nucleomics data. *Nat. Commun.* **13**, 2365 (2022).

102. Chen, Z. *et al.* High-resolution and high-accuracy topographic and transcriptional maps of the nucleosome barrier. *eLife* **8**, e48281 (2019).

103. Ngo, K. X., Kodera, N., Katayama, E., Ando, T. & Uyeda, T. Q. Cofilin-induced unidirectional cooperative conformational changes in actin filaments revealed by high-speed atomic force microscopy. *eLife* **4**, e04806 (2015).

104. Phillips, J. C. *et al.* Scalable molecular dynamics on CPU and GPU architectures with NAMD. *J. Chem. Phys.* **153**, 044130 (2020).

105. Ivani, I. *et al.* Parmbsc1: a refined force field for DNA simulations. *Nat. Methods* **13**, 55–58 (2016).

106. Maier, J. A. *et al.* ff14SB: Improving the accuracy of protein side chain and backbone parameters from ff99SB. *J. Chem. Theory Comput.* **11**, 3696–3713 (2015).

107. Peters, M. B. *et al.* Structural Survey of Zinc-Containing Proteins and Development of the Zinc AMBER Force Field (ZAFF). *J. Chem. Theory Comput.* **6**, 2935–2947 (2010).

108. Jorgensen, W. L., Chandrasekhar, J., Madura, J. D., Impey, R. W. & Klein, M. L. Comparison of simple potential functions for simulating liquid water. *J. Chem. Phys.* **79**, 926–935 (1983).

109. Yoo, J. & Aksimentiev, A. Improved Parametrization of $Li^+$ , $Na^+$ , $K^+$ , and $Mg^{2+}$ Ions for All-Atom Molecular Dynamics Simulations of Nucleic Acid Systems. *J. Phys. Chem. Lett.* **3**, 45–50 (2012).

110. Miyamoto, S. & Kollman, P. A. Settle: An analytical version of the SHAKE and RATTLE algorithm for rigid water models. *J Comput Chem* **13**, 952–962.

111. Andersen, H. C. Rattle: A "velocity" version of the shake algorithm for molecular dynamics calculations. *J. Comput. Phys.* **52**, 24–34 (1983).

112. Darden, T., York, D. & Pedersen, L. Particle mesh Ewald: An N·log(N) method for Ewald sums in large systems. *J. Chem. Phys.* **98**, 10089–10092 (1993).

113. Martyna, G. J., Tobias, D. J. & Klein, M. L. Constant pressure molecular dynamics algorithms. *J. Chem. Phys.* **101**, 4177–4189 (1994).

114. Allen, M. P. & Tildesley, D. J. *Computer Simulation of Liquids*. (Oxford University Press, 2017). doi:10.1093/oso/9780198803195.001.0001.

115. Payne, M. C. Iterative minimization techniques for *ab initio* total-energy calculations: molecular dynamics and conjugate gradients. *Rev. Mod. Phys.* **64**, 1045–1097 (1992).

116. Humphrey, W., Dalke, A. & Schulten, K. VMD: visual molecular dynamics. *J. Mol. Graph.* **14**, 33–38, 27–28 (1996).

117. Gordon, J. C. *et al.* H++: a server for estimating pKas and adding missing hydrogens to macromolecules. *Nucleic Acids Res.* **33**, W368-371 (2005).

118. Case, D. A. *et al.* AmberTools. *J. Chem. Inf. Model.* **63**, 6183–6191 (2023).

119. Gowers, R. J. *et al.* MDAnalysis: A Python Package for the Rapid Analysis of Molecular Dynamics Simulations. *scipy* https://doi.org/10.25080/Majora-629e541a-00e (2016) doi:10.25080/Majora-629e541a-00e.

120. Jensen, S. B. *et al.* Biased cytochrome P450-mediated metabolism via small-molecule ligands binding P450 oxidoreductase. *Nat. Commun.* **12**, 2260 (2021).

121. Bohr, F. *et al.* Enhanced hexamerization of insulin via assembly pathway rerouting revealed by single particle studies. *Commun. Biol.* **6**, 178 (2023).

122. Slot, T. K., Riley, N., Shiju, N. R., Medlin, J. W. & Rothenberg, G. An experimental approach for controlling confinement effects at catalyst interfaces. *Chem. Sci.* **11**, 11024–11029 (2020).

123. Pollard, K. S., Hubisz, M. J., Rosenbloom, K. R. & Siepel, A. Detection of nonneutral substitution rates on mammalian phylogenies. *Genome Res.* **20**, 110–121 (2010).

124. Bell, G. I. Models for the specific adhesion of cells to cells. *Science* **200**, 618–627 (1978).

125. Banigan, E. J. *et al.* Transcription shapes 3D chromatin organization by interacting with loop extrusion. *Proc. Natl. Acad. Sci. U. S. A.* **120**, e2210480120.

126. Garcia, P. *et al.* Disruption of NIPBL/Scc2 in Cornelia de Lange Syndrome provokes cohesin genome-wide redistribution with an impact in the transcriptome. *Nat. Commun.* **12**, 4551 (2021).

## MATERIALS AND METHODS

### *Materials*

See supplementary Microsoft Excel spreadsheet for all materials, buffers, and sequences.

### *Experimental methods*

#### Protein preparation:

#### Human CTCF cloning

The insect cell expression construct for full length human CTCF (pLib 10xHis-CTCF_1-727-Halo-Flag; #Mz92) was previously described[28]. N-terminally deleted human CTCF (10xHis-CTCF_266-727-Halo-Flag; #Mz93) and C-terminally deleted human CTCF (10xHis-Halo-CTCF_1-579-Flag; #Mz51) were generated by PCR using Phusion Hot Start Flex DNA Polymerase (NEB; M0535S) and inserted into pLib[86] using Gibson assembly[87].

#### Human CTCF protein expression and purification

Full length and truncated forms of CTCF were expressed and purified as described[28] with modifications. Briefly, *Spodoptera frugiperda* Sf9 cells derived from pupal ovarian tissue of the female fall armyworm (ThermoFisher Scientific; B82501) were cultured at 100 rpm and 27 $^{o}$C in Grace medium (prepared in-house) supplemented with 10% fetal calf serum (Gibco; A5256801), 2 mM L-glutamine (Gibco; 25030-024), 1x Penicillin-Streptomycin (Sigma-Aldrich; P0781) and 0.1% Poloxamer 188 (Gibco; 24040-032). Bacmids were generated in *E. coli* DH10EmBacY as described[88].

For baculovirus infection of Sf9 cells, $1x10^6$ Sf9 cells were seeded on a 6 well plate in 3 ml Sf-900 SFM media (ThermoFisher Scientific; 12658027), transfected with bacmid DNA using Fugene 6 (Promega) and incubated for 96 hours at 27 °C. The baculovirus-containing cell supernatant (V0) was then collected and used to infect 50 ml Sf9 cells at a density of 1 $x10^6$ cells/ml in Grace medium supplemented as described above. Cells were grown at 100 rpm and 27 °C, centrifuged 72 hours after infection and 9 ml cell supernatant (V1) was then used to infect 750 ml Sf9 cells at a density of 1.2 $x10^6$ cells/ml in Grace medium supplemented as above in the presence of 0.1 mM $ZnCl_2$. Around 4 x 750 ml cultures were infected per construct. Cells were centrifuged around 54 hours after infection, washed in PBS, frozen in liquid nitrogen and stored at -80 °C.

Around 20 ml cell pellet per construct was lysed by Dounce homogenisation and resuspended to 100 ml in CTCF buffer (35 mM HEPES pH 7.5, 350 mM NaCl, 5% glycerol) supplemented with 0.1 mM $ZnCl_2$, 0.05% Tween-20, 5 mM imidazole, 1 mM PMSF, EDTA-free cOmplete tablet (1 per 50 ml) (Roche; 11873580001), 3 mM

betamercaptoethanol, 10 µg/ml aprotinin (Carl Roth; A162.3), 2 mM benzamidine, benzonase (prepared in-house) and DNase I (800 units, prepared in-house). The lysates were cleared by centrifugation at 41,000 g for 45 min at 4°C. The soluble fractions were incubated with 2 ml NiNTA agarose (Qiagen, 30230) for 90 min at 4°C and washed with CTCF buffer supplemented with 0.1 mM $ZnCl_2$, 0.01 % Tween-20, 35 mM imidazole, 1 mM PMSF and 2 mM benzamidine. Proteins were eluted with CTCF buffer supplemented with 0.1 mM $ZnCl_2$, 1 mM PMSF, 2 mM benzamidine and 300 mM imidazole. The eluates were then combined with 2 ml of FLAG-M2 agarose resin (Sigma; A2220) and incubated for 2 hours at 4°C. Beads were washed with Flag buffer 350 (35 mM HEPES pH 7.5, 350 mM NaCl, 5% glycerol) supplemented with 0.1 mM $ZnCl_2$. Beads were resuspended in one bed volume of Flag buffer 350 supplemented with 0.1 mM $ZnCl_2$ and incubated protected from light in the presence of excess JF646-HaloTag Ligand at room temperature with rotation for 20 min. Beads were washed extensively with Flag buffer 350 supplemented with 0.1 mM $ZnCl_2$ and then with Flag buffer 350 in the absence of 0.1 mM $ZnCl_2$. Bound proteins were eluted with Flag buffer 350 supplemented with 0.5 mg/ml 3xFlag peptide. Eluates were concentrated using Vivaspin 20 50 kDa MWCO ultrafiltration units (Sartorius; VS2032), frozen in liquid nitrogen and stored at -80°C.

**Janelia Fluor 646-HaloTag Ligand preparation for CTCF labeling**

A 20 mM stock of Janelia Fluor 646 SE (Tocris; 6148) and a 50 mM stock of HaloTag Amine (O2) Ligand (Promega; P6711) were prepared in dimethylformamide (DMF) immediately prior to use. Aliquots of these stocks were combined, diluted in DMF and supplemented with diisopropylethylamine (final 250 µl reaction mixture: 6.74 mM Janelia Fluor 646 SE, 2.69 mM HaloTag Amine (O2) Ligand, 37 mM diisopropylethylamine). The reaction mixture was incubated at room temperature for ~ 16 hours protected from light. The reaction mixture was diluted 10-fold in solvent A (5 % acetonitrile, 0.1 % formic acid) and purified in five successive reverse phase HPLC runs using an Ultimate 3000 (ThermoFisher Scientific) equipped with a Kinetex 5µ XB-C18 100A, 250 x 4.6mm column using the following gradient of solvents: A (5 % acetonitrile, 0.1 % formic acid), B (acetonitrile, 0.1 % formic acid); 0 – 100 % B in 30 min at a flow rate of 0.8 ml / min. Peak fractions were pooled, lyophilized, resuspended in DMF, frozen in liquid nitrogen and stored at -80°C.

**Human STAG1, SMC1a, SMC3, RAD21 and PDS5A cloning**

Human STAG1 and PDS5A were amplified from human cDNA. Genes were inserted into 438B (Addgene plasmid 55219) by Ligation Independent Cloning (LIC). The genes were inserted behind a 6xHis tag followed by a Tobacco etch virus protease (TEV) cleavage site. msfGFP-PDS5A was generated by inserting a 6xHis-10xArg-msfGFP-TEV tag before the PDS5A coding region.

Human SMC1a and RAD21 were amplified from Addgene plasmids 32363 and 57666, respectively. SMC3 was amplified from human cDNA. SMC1a and SMC3 were inserted into vector 438A. RAD21 was cloned into vector 438F where RAD21 was followed by a TEV cleavage site and a maltose binding protein (MBP) tag. The subunits were combined into pBig1a to create a co-expression vector[86].

Purified plasmid DNA (0.3–0.8 µg) was electroporated into DH10αEMBacY cells to generate bacmids (Geneva Biotech)[89]. DNA was extracted by isopropanol precipitation from positive clones and transfected into Sf9 cells grown in ESF921 (Expression Systems) with X-tremeGENE9 transfection reagent (Sigma). The resulting virus (V0) was harvested 48–72 h after transfection. Virus was then amplified by infecting 25 mL of Sf9 or Sf21 cells with 25–150 µL of the harvested V0, and grown at 27°C, 300 rpm. Viruses were harvested 48–72 h after proliferation arrest by centrifugation and stored at 4°C.

For protein expression, 600 ml of Hi5 cells at 1 million cells/mL confluency were infected with 300-500 µL of virus and grown at 27° for 60-72h in ESF921 medium. The cells were collected by centrifugation. STAG1 was resuspended in Lysis Buffer 1 (STAG1: 300 mM KCl, 20 mM Tris-HCl pH 7.5, 30 mM Imidazole pH 8.0, 5 mM beta mercaptoethanol (BME), 10% glycerol (v/v), 0.284 µg $ml^{-1}$ leupeptin, 1.37 µg $ml^{-1}$pepstatin A, 0.17 mg $ml^{-1}$ PMSF, and 0.33 mg $ml^{-1}$ benzamidine). PDS5A, GFP-PDS5A were resuspended in Lysis buffer 2 (500 mM NaCl, 25 mM Tris-HCl pH 7.5, 30 mM Imidazole pH 8.0, 5 mM BME, 10% glycerol (v/v), 0.284 µg $ml^{-1}$ leupeptin, 1.37 µg $ml^{-1}$pepstatin A, 0.17 mg $ml^{-1}$ PMSF, and 0.33 mg $ml^{-1}$ benzamidine) at 4°, snap frozen in liquid nitrogen, and stored at -80°. SMC1a-RAD21-MBP-SMC3 pellets were resuspended in Lysis Buffer 3 (Lysis Buffer: 500 mM NaCl, 20 mM Tris-HCl pH 7.9, 30 mM Imidazole pH 8.0, 5 mM BME, 10% glycerol (v/v), 0.284 µg $ml^{-1}$ leupeptin, 1.37 µg $ml^{-1}$pepstatin A, 0.17 mg $ml^{-1}$ PMSF, and 0.33 mg $ml^{-1}$ benzamidine) and immediately processed for purification.

#### Human STAG1 and PDS5A/PDS5A-GFP purification

All steps were performed at 4° unless otherwise specified. Frozen cell pellets were thawed in a room temperature water bath and lysed by sonication. Lysates were clarified by centrifugation. The clarified lysate was filtered successively through 5 µm and 0.45 µm syringe filters. Filtered lysate was applied to a 5 mL HisTrap (Cytiva) column equilibrated in Lysis Buffer. The loaded lysate was washed with 10 CV of Lysis Buffer, followed by a 5 CV of High Salt Buffer (STAG1: 1000 mM KCl, 20 mM Tris-HCl pH 7.5, 30 mM Imidazole pH 8.0, 5 mM BME, 10% glycerol (v/v). PDS5A and GFP-PDS5A: 1000 mM NaCl, 25 mM Tris-HCl pH 7.5, 30 mM Imidazole pH 8.0, 5 mM BME, 10% glycerol (v/v)), and 5 CV of Lysis Buffer, followed by 5 CV of Low Salt Buffer (STAG1: 150 mM KCl, 20 mM Tris-HCl pH 7.5, 30 mM Imidazole pH 8.0, 5 mM BME, 10% glycerol (v/v), PDS5A and msfPDS5A 150 mM NaCl, 25 mM Tris-HCl pH 7.5, 30 mM Imidazole pH 8.0, 5 mM BME, 10% glycerol (v/v)). The HisTrap was attached to a HiTrapQ column equilibrated in Low Salt buffer. Protein was eluted from the HisTrap column onto HiTrap Q column over a gradient from

0-100% nickel elution buffer (STAG1: 150 mM KCl, 20 mM Tris-HCl pH 7.5, 500 mM Imidazole pH 8.0, 5 mM BME, 10% glycerol (v/v), PDS5A and msfGFP-PDS5A: 150 mM NaCl, 25 mM Tris-HCl pH 7.5, 300 mM Imidazole pH 8.0, 5 mM BME, 10% glycerol (v/v)). After eluting the protein, the HisTrap column removed. The HiTrapQ column was washed with 5 CV of Low Salt buffer. The HiTrapQ column was developed over a gradient from 0%-100% High Salt Buffer. Peak fractions were analyzed on 10% Tris-Glycine SDS-PAGE. Fractions containing the protein of interest were pooled, mixed with 1.5 mg of His6-TEV protease (except for msfGFP-PDS5A), and dialyzed overnight in 7kDa MWCO SnakeSkin tubing (Fisher) at 4° against 1-2 L of Dialysis Buffer (STAG1: 300 mM KCl, 20 mM Tris-HCl pH 7.5, 30 mM Imidazole pH 8.0, 5 mM BME, 10% glycerol (v/v). PDS5A/GFP-PDS5A: 500 mM NaCl, 25 mM Tris-HCl pH 7.5, 30 mM Imidazole pH 8.0, 5 mM BME, 10% glycerol (v/v)).

Protein was removed and applied to a 5 mL HisTrap column (except for msfGFP-PDS5A, which was directly used for size exclusion chromatography), equilibrated in the appropriate Lysis Buffer. This enabled the capture of the 6xHis-MBP tag, 6xHis-TEV protease, and uncleaved protein. The cleaved protein was collected as flow through and concentrated in a 30K MWCO 15 mL Amicon Millipore ultrafiltration device. The concentrated protein was then applied to a STAG1: Superose 6 increase column or an S200 16/600 HiLoad column (Cytiva) or PDS5A/GFP-PDS5A: Superose 6 10/300 pg increase column (Cytiva) equilibrated in Size Exclusion Chromatography Buffer (STAG1: 300 mM KCl, 20 mM Tris-HCl pH 7.5, 0.5 mM Tris(2-carboxyethyl)phosphine (TCEP), 10% glycerol (v/v), PDS5A/GFP-PDS5A: 500 mM NaCl, 25 mM Tris-HCl pH 7.5, 0.5 mM TCEP, 10% glycerol (v/v)). Peak fractions were analyzed on 10% Tris-Glycine SDS-PAGE. Appropriate fractions were pooled and concentrated in a 30K MWCO Amico Millipore ultrafiltration device. Protein was aliquoted, snap frozen in liquid nitrogen, and stored in -80°.

**Human Cohesin (SMC1a, SMC3, RAD21) purification**

Hi5 cells infected with cohesin virus were harvested after 60-65h of infection and were immediately processed for protein purification. Cells were collected by centrifugation at 238xg for 30 minutes. Cell pellets were resuspended in Lysis Buffer and lysed by sonication. Lysed cells were clarified by centrifugation. The clarified lysate was filtered successively through 5 μm and 0.45 μm syringe filters. Once filtered, the lysate was applied to a custom amylose column packed with 10-15 mL of Amylose resin (New England Biolabs), equilibrated in Lysis Buffer. The loaded lysate was washed with 2 CV of Lysis Buffer, followed by a 2 CV High Salt wash (1000 mM NaCl, 20 mM Tris-HCl pH 7.9, 30 mM Imidazole pH 8.0, 5 mM BME, 10% glycerol (v/v)), and 2 CV of Lysis Buffer. Protein was eluted with 2 CV of Amylose Elution Buffer (500 mM NaCl, 20 mM Tris-HCl pH 7.9, 300 mM Maltose, 5 mM BME, 10% glycerol (v/v)). Peak fractions were analyzed by 6% Tris-Glycine SDS-PAGE and Coomassie Staining. Appropriate fractions were pooled and concentrated in a 15 mL 50K MWCO Amicon Millipore ultrafiltration device. Concentrated protein (1-1.5 mL) was loaded on a Superose 6 10/300 increase column (Cytiva), equilibrated in Size Exclusion Chromatography Buffer (500 mM NaCl, 20 mM Tris-HCl pH 7.9, 0.5 mM TCEP, 10% glycerol (v/v)). Peak fractions were analyzed on 10% Tris-Glycine SDS-PAGE and coomassie staining. Appropriate fractions were pooled and concentrated in a 50K MWCO Amicon Millipore ultrafiltration device. Protein was aliquoted, snap frozen in liquid nitrogen, and stored in -80°.

**Mouse CTCF protein expression and purification**

The mouse fusion protein 3X-FLAG-Halo-TEV-mCTCF-HisX6 recombinant Bacmid DNA[90] was generated using the Bac-to-Bac Baculovirus expression system (ThermoFisher 10359016). Sf9 cells were infected with the baculovirus to express the recombinant fusion protein. The recombinant protein was purified from Sf9 cells, as previously described[90]. Briefly, the cell culture was centrifuged to isolate the cell pellet. The cell pellet was gently resuspended and washed with PBS solution and centrifuged at 3,900 x g for 10 minutes. The cell pellet was gently resuspended in five packed cell volumes of a mouse CTCF high salt buffer (1 M NaCl, 50 mM HEPES pH 7.9, 0.05% NP-40, 10% glycerol, 10 mM 2-mercaptoethanol) and sonicated. The solution was centrifuged for 30 min at 12,000 rpm. The clarified lysate was supplemented with 10mM imidazole pH 8.0. The sample was transferred and incubated with an equilibrated NI-NTA resin (Qiagen 30410) on a rocker for 90 minutes. The beads were washed several times with four column volumes of mouse CTCF high salt buffer supplemented with 20mM imidazole, followed by washing the beads twice with four column volumes of 50 mM HEPES pH 7.9, 10% glycerol, 0.01% NP-40, and 20 mM imidazole. The protein was eluted with 0.5 M NaCl, 50 mM HEPES pH 7.9, 10% glycerol, 0.01% NP-40, and 0.25 M imidazole. The eluted fractions were analyzed by SDS-PAGE gel followed by SimplyBlue SafeStain (Invitrogen LC6060). Selected elution fractions containing protein were combined and transferred to an equilibrated Anti-FLAG M2 Affinity gel (Millipore Sigma A2220) and incubated on a rocker for 3 hours. The resin was washed with 1 M NaCl, 50 mM HEPES pH 7.9, 0.05% NP-40, and 10% glycerol and then equilibrated and washed with 0.2 M NaCl, 50 mM HEPES pH 7.9, 10% glycerol, and 0.01% NP-40. The protein was eluted with 0.4 mg/mL 3XFLAG peptide (Millipore Sigma F4799) in 0.2 M NaCl, 50 mM HEPES pH 7.9, 10% glycerol, and 0.01% NP-40 buffer. Protein concentrations were determined using an SDS PAGE gel stained with ProtoStain Blue Colloidal Coomassie Stain (National Diagnostics EC-727) and were compared to Bovine Serum Albumin (BSA) standards. All protein purification steps were performed at 4℃ and in the presence of protease inhibitors (or cOmplete EDTA-free Protease Inhibitor Cocktail (Roche 11836170001)).

**Cloning, expression and purification of the NIPBL and MAU2 complex**

*H. sapiens* NIPBL and MAU2 were amplified from cDNA and cloned into the MacroLab 438 vector series for expression in insect cells using ligation-independent cloning[91].The NIPBL expression construct contained an N-terminal 3x FLAG tag, followed by a Halo tag, and a HRV 3C protease cleavage site, and a C-terminal TEV protease cleavage site, followed by a 10x His tag. MAU2 was cloned into the 438-A vector with no tag. Both subunits were then combined into the pBigBac1b vector[86].Plasmids were sequence verified by long-read sequencing. Bacmid, virus, and protein production were then performed as previously described[92]. The NIPBL/MAU2 complex was expressed in Hi5 insect cells and purified at 4°C. Cells expressing NIPBL/MAU2 were resuspended in lysis buffer (250 mM NaCl, 20 mM Na HEPES pH 7.4, 10% (v/v) glycerol, 30 mM imidazole, 1 mM TCEP, 0.284 µg $ml^{-1}$ leupeptin, 1.37 µg $ml^{-1}$ pepstatin A, 0.17 mg $ml^{-1}$ PMSF and 0.33 mg $ml^{-1}$ benzamidine) and lysed by sonication. The lysate was centrifuged and cleared by ultra-centrifugation. The supernatant containing NIPBL/MAU2 was filtered using 0.22 µm syringe filters. The filtered supernatant was applied to a HisTrap HP 5 mL column (Cytiva 17524801) previously equilibrated in lysis buffer. The column was subsequently washed with 10 CV of lysis buffer and 5 CV of low salt buffer (150 mM NaCl, 20 mM Na·HEPES pH 7.4 at 25 °C, 10% (v/v) glycerol, 1 mM TCEP). The NIPBL/MAU2 complex was eluted onto a 5 mL HiTrap Q (Cytiva 17115401) with 10 CV low salt buffer supplemented with 500 mM Imidazole. The HisTrap column was removed, and the HiTrap Q column was washed with 10 CV of low salt buffer. The NIPBL/MAU2 complex was eluted with a 20 CV linear gradient with high salt buffer (1 M NaCl, 20 mM Na·HEPES pH 7.4 at 25 °C, 10% (v/v) glycerol, 1 mM TCEP). The elution was fractionated and analyzed using SDS-PAGE. Fractions containing NIPBL/MAU2 were pooled and concentrated using an Amicon 100,000 MWCO centrifugal filter unit (Millipore UFC910008). The concentrated sample was applied to a Superose 6 Increase 10/300 GL (Cytiva 29091596) column equilibrated in gel filtration buffer (250 mM NaCl, 20 mM Na·HEPES pH 7.4,10% (v/v) glycerol, 1 mM TCEP pH 8). NIPBL/MAU2-containing fractions were concentrated with an Amicon 100,000 MWCO centrifugal filter unit, aliquots were frozen in liquid nitrogen and stored at −80 °C. Throughout the manuscript, the NIPBL/MAU2 complex will be referred to as “NIPBL”.

**Construct preparation:**

**Unzipping construct preparation for optical tweezers experiments**

The “Y-shaped” DNA constructs for single-molecule unzipping experiments (DNA handles) were generated as described previously[47,59,60] with minor modifications. Two 2000 bp dsDNA handles were PCR-amplified from lambda DNA (NEB N3011) using Q5 DNA High-Fidelity Polymerase (NEB M0491S) to include restriction sites for 3×digoxigenin and 3×biotin tag ligation (to improve stability under force)[81] as well as sites for nicking enzymes. The handles were treated with Nt.BbvCI (NEB R0632L) and Nb.BbvCI (NEB R0631L) restriction enzymes to produce complementary ~29-nt overhangs, which were annealed in equimolar ratios to form a ~4000 bp construct in annealing buffer (1x TAE (40 mM Tris-acetate, 10 mM EDTA), 12.5 mM MgAc). A ~230 bp alignment sequence was PCR-amplified from 601c2-plasmid[81] with Q5 polymerase, restriction digested with DraIII-HF (NEB R3510S), and ligated to the construct with T4 DNA ligase (NEB M0202M), which was then size selected with gel-excision from E-gels (Thermo Scientific G402021, G402022, G401004) using a Qiaquick Gel Extraction Kit (Qiagen 28704). The digoxigenin- and biotin-modified oligos were ligated to overhangs generated by BglI (NEB R0143S) and underwent DraIII-HF restriction digestion, respectively. Variable sequences (500-1000 bp), including the CTCF consensus sequence, the *PDGFRA* insulator, and the *PDGFRA* insulator fused with a Widom-601 nucleosome positioning sequence, were restriction digested with BbsI-HF (NEB R3539L) to ligate a short DNA hairpin with T4 DNA ligase, and DraIII-HF to create a complementary overhang for purified DNA handles. All PCR, restriction digestion, and ligation reactions were subsequently purified with a Qiaquick PCR Purification Kit (Qiagen 28106) or CleanNGS beads (Bulldog Bio CNGS050). Variable regions contained a single CTCF binding site and were otherwise computationally designed to minimize off-target binding. The CTCF binding sites used were the *PDGFRA* motif (+/- Widom 601 sequence), the consensus motif, or an alternative consensus motif with slight variation in the periphery used for mouse CTCF experiments.

For optical tweezers experiments, the final construct was assembled by mixing Rapid Ligation Buffer (Promega C6711), prepared DNA handles (1 nM final), variable region (20-30 nM final), 1 uL ~2.08 μm, anti-digoxigenin-modified, polystyrene beads (Spherotech DIGP-20-2), 1 uL concentrated T4 DNA ligase (NEB M0202L), and nuclease free water to a final volume of 10 uL. This mixture was incubated at RT for 10 min and diluted to a working concentration by adding 500 uL of experimental buffer.

**CpG Methylation of *PDGFRA* constructs**

For methylation experiments, 400 ng of the fully-prepared *PDGFRA* unzipping variable region was treated with 1 uL of M.Sssi (NEB M0226S) and 13 uM S-adenosyl-methionine (SAM) (NEB B9003S) following the manufacturer's protocol. The treatment was validated by restriction digestion with HinP1I (NEB R0124S), which is ineffective at digesting methylated DNA. By running an E-gel of the reaction product against an unmethylated positive control, we determined that the vast majority of the sample was protected from digestion and therefore methylated.

**Lambda DNA for confocal C-trap and TIRF measurements**

Lambda DNA (NEB N3011S) containing 12-nucleotide 5′ overhangs were filled using Klenow fragment lacking exonuclease activity (NEB M0212S). The polymerization reaction included 16 nM lambda DNA, 33 μM each of dATP (Qiagen 201912), dGTP (Qiagen 201912), biotinylated-dUTP (ThermoFisher R0081), and biotinylated-dCTP (ThermoFisher 19518018), and 3 μL of Klenow in a 50 μL final volume with NEB buffer 2. The mixture was incubated at 37 °C for 1 hour and heat-inactivated at 75 °C for 20

minutes. To remove excess nucleotides and proteins, drop dialysis was performed using 0.025 µm VSWP filters (Millipore Sigma VSWP02500) suspended over 20–25 mL of dialysis buffer (10 mM Tris-HCl, pH 8.0; 0.1 mM EDTA) in a petri dish. A 50–150 µL drop of DNA was gently placed on the membrane using a large-orifice pipette tip, ensuring the membrane did not dip into the buffer. Dialysis proceeded at room temperature for at least 1.5 hours, after which the DNA was carefully retrieved and stored at 4 °C for long-term use.

**HS-AFM sample preparation**

A nearly symmetric, ~8 kb (7,979 bp; including 3 ssDNA bases from RE digestion on both ends) DNA substrate containing consensus CTCF motifs near each end (centered 514 or 511 bp from the end) was ordered within a pBluescript II KS(+) plasmid vector from GenScript Biotech. The plasmid was amplified through transformation into JM109 competent cells (Promega L2005), plating on LB agar plates with ampicillin (Teknova L1004), inoculation in LB media (ThermoFisher 12795027) containing 100 ug/mL ampicillin (Sigma-Aldrich A9518-100G), and purification with the E.Z.N.A.® Plasmid Mini Kit II (Omega Bio-Tek D6945-02). The substrate was then removed from the backbone with DraIII restriction enzyme digestion, as described above, and subsequently size selected with gel-excision purification, as described above. A ~1 kb DNA (1,006 bp) segment of the 8 kb DNA substrate with a central CTCF consensus motif (510 bp and 495 bp from motif center on each side) was prepared using PCR, as described above, with the 8 kb DNA as a template.

**Nucleosome reconstitution**

The variable region containing a Widom-601 positioning sequence was prepared following the same protocol as other variable regions. Once amplified, restriction digested, and hairpin-ligated, ten titrations of different Octamer:DNA ratios were prepared (0.50x, 0.75x, 1.00x, 1.25x, 1.50x, 1.75x 2.00x, 2.50x, 3.00x, and 4.00x), with 1 pmol of DNA each and an increasing pmol of human recombinant octamer (EpiCypher 16-0001). Each titration was brought to 40 uL volume using a high salt buffer (2M NaCl, 10mM Tris-Cl pH 7.5, 1mM EDTA, 3.5 mM BME, 0.02% NP40)[93]. Reaction solutions were transferred to mini dialysis tubes (ThermoFisher 69560) for dialysis in 600 mL of high salt buffer. Buffer exchange was achieved using a peristaltic pump that slowly transferred low salt buffer (50mM NaCl, 10mM Tris-Cl pH 7.5, 1mM EDTA, 3.5 mM BME, 0.02% NP40) into the dialysis beaker for 18 hours at 4°C. Nucleosomes were harvested and their presence was confirmed using 6% DNA retardation gel (ThermoFisher EC63655) by loading 10 uL of sample with 2uL 6X purple loading dye with no SDS (NEB B7024), at 110 Volts, using 0.5X TBE, for 1 hour. The gel was stained with SYBR Safe (ThermoFisher S33102) and imaged.

**Cell culture**

HEK293T (ATCC® CRL-3216) cells were maintained at 37 °C with 5% $CO_2$ in Dulbecco's Modified Eagle Medium (DMEM) (Corning 10-013-CM) supplemented with 10% fetal bovine serum (FBS) (Clontech Labs 631106), 100 units/mL Antibiotic-Antimycotic (100X) (Thermo Fisher Scientific 15240112) (referred to as DMEM complete). Routine mycoplasma testing was performed monthly.

### Optical tweezers experiments:

#### Optical tweezers conditions and sample capture for unzipping experiments

Imaging was performed using a commercial optical tweezers setup, the C-Trap® (LUMICKS). Calibration of the setup was carried out by analyzing the thermal fluctuations of trapped beads, sampled at 78,125 Hz, with a typical stiffness of ~0.2 pN/nm. Before measurements, the laminar flow-based microfluidics system was passivated by flowing bovine serum albumin (BSA; 0.1% wt/vol in phosphate-buffered saline (PBS)) for 30 minutes, followed by a 30-minute PBS flush. The trapping laser was set to 100% laser power, with overall power at 30% and Trap 1 split power at 70%, achieving ~0.2-0.35 pN/nm stiffness for both traps. Tether formation was performed *in situ* (inside the flow cell) by trapping an anti-digoxigenin bead (bound by DNA handles ligated to CTCF motif-containing construct) in one trap, trapping a ~4.34 µm, streptavidin-coated polystyrene bead (Spherotech SVP-40-5) in the second trap, and bringing the two beads into close proximity to allow binding of the 3×biotin tag in the DNA handles to the streptavidin on the large bead.

#### Unzipping DNA with optical tweezers

In unzipping experiments, after forming a DNA tether, the distance between the traps was linearly increased at 0.6 µm/s (unless otherwise noted) until the terminal hairpin of the tethered construct was stretched to ~25-30 pN. For CTCF unzipping, the tethered beads were incubated and unzipped in a channel containing 3-10 nM WT, ΔN, or ΔC human CTCF or WT mouse CTCF in 1x CL100 buffer[28] (35 mM Tris pH 7.5, 100 mM KCl, 5 mM $MgCl_2$, 5% Glycerol, 0.005% Tween-20, 0.1 mg/mL BSA, 1 mM TCEP) or CL100* buffer[28] (35 mM Tris pH 7.5, 50 mM KCl, 50 mM NaCl, 5 mM $MgCl_2$, 5% Glycerol, 0.003% Tween-20, 0.1 mg/mL BSA). No statistically significant differences were observed in rupture forces for the two buffers (K-S test $p > 0.05$).

For unzipping experiments involving cohesin-STAG1, cohesin and STAG1 were pre-incubated at an equimolar ratio for 10 minutes on ice at final concentrations of 50 nM in 1x CL100* buffer. When unzipping CTCF-PDS5A-cohesin-STAG1 or CTCF-NIPBL-cohesin-STAG1, the 50 nM preformed cohesin-STAG1 complex was then additionally pre-incubated with PDS5A and/or NIPBL at an equimolar ratio for 10 minutes on ice at final concentrations of 45 nM in 1x CL100* buffer.

When unzipping CTCF in the presence of cohesin complexes or subunits, the tethered beads were incubated and unzipped in a channel containing 10-20 nM of each component at equimolar ratios in 1x CL100* buffer. When NIPBL was used, the flowed-in buffer and protein mixture was preheated to 37C, constantly replenished, and spiked with 5 mM ATP.

**CTCF unzipping survival experiments**

For survival experiments, after incubation with CTCF (and verification of binding by “poking” the protein without rupture of protein:DNA interactions), the beads were moved to a protein-free channel prior to cyclical unzipping. In the “low tension” survival condition, the construct was only partially unzipped to just past the observed CTCF rupture force peak and then rezipped. Cyclical unzipping was performed until the characteristic force peak was not observed for 3 cycles upon which the last cycle the protein was detected in was recorded. Detection of bound proteins was based on an increase in force exceeding 0.5 pN above the median force at the same position in experiments without CTCF.

**Unzipping specificity control**

To quantify the specificity of CTCF on- vs off-targets under unzipping experimental conditions, we cyclically unzipped and rezipped DNA handles ligated to the consensus CTCF construct in a microfluidics channel containing 5 nM CTCF in 1x CL100 buffer and counted the number of peaks observed at the known, expected location and other off-target locations. Percentages were calculated based on the number of unzipping cycles containing, or lacking, each classification of rupture force peak. A total of 159 cycles across 19 replicates were collected from 6 different DNA tethers.

**CTCF-dCas9 competition in optical tweezers**

dRNPs (nuclease-deficient ribonucleoprotein) were prepared to 12 uM by mixing 0.5 uL of 62 uM S.p. dCas9 (IDT 1081067), 0.9 uL of 50 uM tracrRNA (IDT 1072534) annealed in Duplex Buffer (IDT 11-01-03-01) to the relevant crRNA (IDT), and 1.1 uL of Cas9 dialysis buffer (20 mM HEPES pH 7.5, and 500 mM KCl, 20% glycerol) and incubating for 10 min at room temperature.

For competition experiments, the handles were incubated in 3 nM CTCF until bound (validated by “poking” as described above), moving to a channel containing 16 nM dRNP, waiting 1 minute, and unzipping to determine the bound protein based on characteristic rupture force. For dissociation controls, the same protocol was followed, but with the 1-minute wait step in the absence of protein. For the dCas9 binding control, the same protocol was performed but beginning in a protein-free channel prior to dCas9 incubation.

**Dynamic measurements using DNA fluctuations**

To measure the dynamics within the CTCF-DNA complex, DNA was partially unzipped until it approached the location of the CTCF binding site, which was known from sequence design and prior unzipping experiments. Fluctuation experiments were possible at reproducible, simulatable, sequence-dependent peaks within the naked DNA unzipping signature (Fig. S1C), which spontaneously thermally fluctuated between ‘open’ and

'closed' states[47,52].The location and magnitude of these peaks result from the underlying sequence of approximately 20-40 bps[52]. The applied force was finely adjusted to achieve an approximately equivalent probability of closed versus open configurations and thus position the fork at a nearly identical location between replicates. The degree of fork openness was titrated and verified to not affect CTCF dynamics in Figure S7.

To measure CTCF dynamics, the construct was moved to a channel containing 3-10 nM WT, ΔN, or ΔC CTCF in 1x CL100. Once protein binding stabilizes the closed state, dynamics were measured for 1-5 minutes. When measuring CTCF dynamics in the presence of cohesin complexes or subunits, the construct was moved to a channel containing 10-20 nM of each component at equimolar ratios in 1x CL100* buffer. When NIPBL was used, the flowed-in buffer and protein mixture was preheated to 37C, constantly replenished, and spiked with 5 mM ATP. Cohesin complexes were prepared as described above for unzipping experiments.

**Testing force-dependence of DNA fluctuation dynamic measurements**

To determine force was affecting the fraction of time spent in each state, we performed a control experiment to one conducted in a previous work[52]. This past work observed no force-dependency on ZF association and dissociation in the regime measured using the same DNA fluctuation scheme.

In the experiment, rather than striving for an approximately even fraction of time spent in the open and closed DNA states prior to CTCF binding, we slightly varied the location of the unzipping fork, which altered the average force felt by the pre-bound complex, and thus the initial degree of openness of the consensus motif DNA. We performed 14 replicates ranging from 22% to 85% unbound open DNA fraction (Fig. S7, and supplementary methods). The same HMM analysis was then performed on the resulting data to examine the fraction of time spent in each state as a function of the unbound force.

**Confocal C-trap measurements**

Tracking of CTCF and PDS5A was performed on a LUMICKS C-Trap® optical-tweezers instrument equipped with laminar-flow microfluidics and confocal fluorescence imaging. The continuous-wave tweezers were operated in a dual-trap regime. A five-channel laminar-flow cell was used with the following contents: (i) streptavidin-coated beads, (ii) biotinylated λ-DNA in CL100*, (iii) imaging buffer (CL100* supplemented with an oxygen scavenging system containing 0.8% w/v dextrose, 2mM Trolox (Santa Cruz Biotechnology 53188-07-1), 1mg/ml glucose oxidase (Sigma-Aldrich G2133-50KU), and 500U/ml catalase(Millipore Sigma 9001-05-2)), (iv) CTCF–JF646 (10 nM), and (v) PDS5A–GFP (10 nM), all at room temperature. Two ~4.34 μm streptavidin-coated polystyrene beads (Spherotech SVP-40-5) were trapped, calibrated, and used to capture λ-DNA, which was then stretched to 5 pN and held at constant force. A worm-like chain model was used to verify dsDNA (contour length 16.49 µm for 48.5 kb, persistence length 46 nm, stretch modulus 1000 pN). DNA was then exposed to CTCF. For CTCF-JF646 imaging, confocal settings were as follows: excitation at 488nm (blue) or/and 638 nm (red) pixel size 100 nm, and pixel dwell time was varied between 0.05-0.3 ms. For PDS5A measurements, DNA with pre-bound CTCF was transferred to the PDS5A–GFP channel for two-color imaging.

### High-speed atomic force microscopy (HS-AFM)

#### Specifications of HS-AFM Microscope

A commercial Sample-Scanning High-Speed Atomic Force Microscope (SS-NEX Ando model) from RIBM (Research Institute of Biomolecule Metrology Co., Ltd.) was used for experiments involving CTCF and cohesin complexes. Tapping mode was employed to minimize interference with the deposited sample, and all deposited samples were captured in solution. Ultra-Short Cantilevers (NanoAndMore USC-F1.2-k0.15-10), specifically designed for high-speed AFM with a resonance frequency of 1200 MHz, a spring constant of 0.15 N/m, and a length of 7 µm, were purchased from NanoAndMore and utilized in these experiments. A wide scanner was employed with scan speeds ranging from 0.05 to 1 frame per second, with the resolution set to 200 × 200 pixels.

#### HS-AFM conditions and sample deposition

Sample was prepared for AFM deposition by incubating 0.2 nM DNA substrate (~8 kb or ~1 kb) with combinations of the following proteins, depending on the experiment performed, in SMC buffer (50 mM HEPES pH 7.5, 100 mM NaCl, 10 mM MgCl2) and incubating at 37C for 5 minutes. Protein concentrations are as follows: CTCF (1 nM final), cohesin-STAG1 (1.5 nM final; pre-incubated as described above), PDS5A (1 nM final), NIPBL (1 nM final), and ATP (5 mM final)

Freshly cleaved mica surfaces (NanoAndMore 50-S-15-15) were coated with 3 uL of poly lysine (P-lysine) (Millipore Sigma 25988-63-0) at a concentration of 0.05 mg/mL. After 3 minutes, the surfaces were washed twice with 80 uL water. 3 uL of sample was then added and incubated for 5-10 minutes. Sample was washed once with and imaged in water.

### Single-molecule total internal reflection microscopy (smTIRF)

#### smTIRF imaging setup and preparation smTIRF was performed using a prism-based Nikon Eclipse Ti microscope. Solid-state lasers Coherent (637nm) and Shanghai Dream Laser Technology (543nm) were used to illuminate JF646 and Sytox Orange dyes, respectively, and fluorescent emission was collected using a water immersion 60X/1.27 NA objective. Laser signal was blocked using a custom filter. An electron-multiplying charge-coupled device (Andor iXon 897) was used to capture images. Movies were recorded using the home-built smCamera 2.0 software.

**smTIRF pulldown of CTCF on ds- and ssDNA constructs**

Methoxy polyethylene glycol (PEG) (Laysan Bio MPEG-SVA-2000-5g) passivated slides containing 2% biotin-PEG (Laysan Bio KIT-BIO-PEG-SVA-5K-mPEG-SVA-5K) were prepared and assembled into flow channels following previously established method[94]. Chambers were functionalized with 0.2 mg/mL neutrAvidin (ThermoFisher 31000) in 1x T50 buffer (10 mM TrisHCl pH 8.0, 50mM NaCl) for 1 minute and subsequently washed with 1x T50 buffer. All biotinylated DNA constructs were diluted to 50nM in 1x CL100* and incubated for 2 minutes. Free DNA molecules were washed off with 1x CL100* buffer. JF646-labeled CTCF was diluted to 75pM in Imaging buffer (1x CL100* buffer, supplemented with an oxygen scavenging system described above) and flowed into the chambers. After 10 minutes, a total of 15 short movies (20 frames of 100 ms exposure at 640nm excitation) were taken for each condition and the number of bound CTCF was quantified. Longer movies were recorded until all JF646 spots photobleached (typically 600 to 2500 frames of 100 ms exposure at 640nm excitation) to determine the number of CTCF monomers. Titration curves for DNA constructs were generated by adding additional CTCF in a single channel with increasing concentrations of JF646-CTCF (0, 5, 10, 25, 50, 75, and 100 pM). Each concentration was incubated for 10 minutes before imaging.

**Lambda DNA TIRF loop extrusion**

TIRF microscope slides were prepared and passivated as defined above and previously described[94], but with chambers containing one inlet and two outlets in a configuration that enabled switching between parallel and perpendicular flow. Sample immobilization was achieved by successively flowing, 100 uL of 1x DNA buffer (20 mM Tris pH 7.5, 150 mM NaCl, 0.25 mg/mL BSA) at 1 mL/min, 40 uL 0.2 mg/mL neutrAvidin in 1x T50 buffer at 1 mL/min (1 minute incubation), and 100 uL 1x DNA buffer at 1 mL/min in parallel and perpendicular configurations. 50 uL of 33 pM biotinylated λ-DNA (prepared as described above) was then added into the channel in 1x DNA buffer at 40 uL/min using parallel flow. The chamber was washed once with 40 uL 1x DNA buffer and once with 40 uL 37C 1x IB (40 mM Tris pH 7.5, 30 mM KCl, 2.5 mM MgCl2, 0.5 mg/mL BSA, 1 mM DTT, 250 nM Sytox Orange (ThermoFisher S11368), supplemented with an oxygen scavenging system described above). Immobilized λ-DNA was imaged using perpendicular flow prior to the addition of protein mix. 50 uL of 2 nM pre-incubated Cohesin-STAG1, 2 nM NIPBL, 5 mM ATP in 1x IB at 37C was added at 40 uL/min with perpendicular flow. The sample was then incubated for 2 minutes without flow, and the same field of view was imaged with and without 40 uL/min perpendicular flow using 1x IB at 37C.

**Sequencing-based methods**

**CTCF-CUT&RUN**

CUT&RUN experiments were performed following the EpiCypher CUT&RUN Protocol[95] with some modifications. 0.5-1 million HEK293T cells were washed twice in CUT&RUN wash buffer (20 mM HEPES, pH 7.5, 150 mM NaCl, 0.5 mM spermidine, 1x cOmplete EDTA-free Protease Inhibitor (Roche 11836170001)) and centrifuged at 600 × g at 4°C for 3 minutes. The cells were resuspended in 150 µl of wash buffer and conjugated to 10 µl of Concanavalin A-coated magnetic beads (Bangs Labs BP531). After 10 minutes at room temperature, the supernatant was removed, and the cells were permeabilized on ice with 50 µl of antibody reaction buffer (CUT&RUN wash buffer + 0.01% digitonin + 2 mM EDTA). Five minutes later, 1 µl of CTCF CUT&RUN-grade CTCF antibody (Cell Signaling D31H2) was added, and the mixture was incubated at 4°C overnight.

The material was washed twice with 200 µl of permeabilization buffer (CUT&RUN wash buffer + 0.01% digitonin) and resuspended in 50 µl of the same buffer, after which 2.5 µl of pAG-MNase (EpiCypher 15-1116) was added and incubated at room temperature for 10 minutes. After washing twice with 200 µl of permeabilization buffer, the samples were resuspended in 50 µl of permeabilization buffer, and 1 µl of 100 mM $CaCl_2$ was added on ice to activate the pAG-MNase. pAG-MNase digestion was performed at 4°C for 2 hours. The reaction was stopped by adding 33 µl of 2× CUT&RUN Stop buffer (340 mM NaCl, 20 mM EDTA, 4 mM EGTA, 10 µg/ml RNase A (NEB T3018L), 50 µg/ml glycogen (Milipore-Sigma 361507) and 1 µl of E. coli spike-in DNA (0.5 ng/µl) (EpiCypher 18-1401) to each tube. CTCF-bound DNA was released and collected after incubation at 37°C for 10 minutes.

The DNA was purified using the Monarch Spin PCR & DNA Cleanup Kit (NEB T1130L), eluted in 12 µl of elution buffer, and used for library preparation with the CUTANA™ CUT&RUN Library Prep Kit (EpiCypher 14-1001) according to the manufacturer's instructions. To avoid adapter carryover, gel extraction was performed using the Qiagen Gel Purification Kit (Qiagen 28704). Sequencing was performed sequenced at the Johns Hopkins Genomics Core Facility on an Illumina NextSeq500 or NovaSeq platform (50-bp paired-end). The sequencing data was analyzed using nf-core/cutandrun[96].

**MNase-seq**

0.5 million HEK293T cells were crosslinked with 1% formaldehyde for 5 minutes, quenched with 130 mM glycine, collected, pelleted for 3 minutes at 600 x g, and resuspended in 100 µL of CUT&RUN wash buffer. Pellets were spun down for 3 minutes at 600 g with a total of two washes.

Next, cells were permeabilized with CUT&RUN wash buffer supplemented with 0.01% (v/v) digitonin and incubated on ice for 5 minutes, after which $CaCl_2$ was added to a final concentration of 6 mM. In total, 1.8 U of MNase (NEB M0247S) was added, and the pellets were incubated for 15 minutes at 37°C. The reaction was stopped by mixing it with 100 µL of CUT&RUN Stop buffer. The material was pelleted for 10 minutes at 14,000 g at 4°C, and the supernatant containing nucleosomal DNA was purified using a MiniElute PCR purification kit (Qiagen 28006).

For library preparation, MNase-digested DNA was purified using AMPure XP beads (Beckman Coulter A63881). A total of 500 ng of purified DNA was used for library preparation with the NEBNext Ultra Library Preparation Kit (NEB E7645L), with 9 PCR cycles. Libraries were analyzed and quantified using an E-Gel and Qubit Fluorometer and sequenced at the Johns Hopkins Genomics Core Facility on the Illumina NovaSeq SP100 (50 bp paired-end).

MNase-seq data was mapped to the hg38 genome and processed using an integrated analysis pipeline for MNase-seq, nf-core/mnase[96,97]. Nucleosome positions were called using DANPOS2[98] as part of the nf-core/MNase-seq pipeline.

**Cas9 activity in permeabilized cells**

250,000 HEK293T cells were collected and centrifuged for 3 minutes at 600 × g. The cells were permeabilized with CUT&RUN wash buffer supplemented with 0.01% (v/v) digitonin and incubated on ice for 5 minutes. The material was washed once in 200 µl of Tween-Wash buffer (0.1% Tween-20, 20 mM HEPES-potassium hydroxide, pH 7.5, 150 mM NaCl, 0.5 mM spermidine, 1 Roche cOmplete EDTA-free tablet per 50 ml buffer, and 0.1% BSA) by centrifugation at 500 × g for 3 minutes. The supernatant was removed, and the pellet was resuspended in 100 µl of *in situ* reaction buffer (15 mM Tris, pH 8.0, 60 mM KCl, 1.5 mM $MgCl_2$, 0.1% BSA).

A Cas9-gRNA RNP complex was assembled, as described above for dCas9 but using catalytically active S.p. Cas9 (IDT 1081059), and added to the cells at a final concentration of 180 nM and incubated for 10 minutes at room temperature. The reaction was stopped by adding 20 µl of Proteinase K (NEB P8107S) and 200 µl of lysis buffer from the DNeasy Blood & Tissue Kit (Qiagen, 69504). Genomic DNA (gDNA) was purified according to the manufacturer's instructions. A total of 40 ng of gDNA was used as a template for qPCR reactions, which were performed using Luna® Universal qPCR Master Mix (NEB M3003E) following the manufacturer's instructions. The data were normalized to the Cas9-untreated control.

**Chromatin GpC methylation, DNA extraction, library prep, and Nanopore sequencing**

HEK293T cells were grown as described previously and collected by trypsinization. GpC methylation was performed as described[71], with modifications. Nuclei were extracted by incubating on ice for 4 min in lysis buffer prepared 1× RSB (100 mM Tris-Cl pH 7.4, 100 mM NaCl, 30 mM $MgCl_2$) and 0.25% NP-40. Intact nuclei were pelleted (5 min, 1,000 g, 4 °C), resuspended in 1× RSB, and pelleted again (5 min, 800 g, 4 °C). For labeling, ~$5\times10^5$ nuclei were combined in 500 µL containing 300 mM sucrose, 1× M.CviPI reaction buffer, 96 µM S-adenosyl-methionine (SAM) (NEB B9003S), and 200 U M.CviPI (NEB M0227L). Reactions were incubated at 37 °C, 1,000 rpm for 7.5 min; SAM was replenished to 96 µM at 7.5 min and incubation continued for 7.5 min. Reactions were stopped with 515 µL pre-warmed stop solution (20 mM Tris-Cl pH 7.9, 600 mM NaCl, 1% SDS, 10 mM EDTA). DNA was purified using the Monarch HMW DNA Extraction Kit (NEB T3050L) following the manufacturer's instructions. Purified gDNA was sheared to ~15 kb using a Covaris g-TUBE (Covaris 520079), quantified by Qubit, and taken forward to library prep. Libraries for Oxford Nanopore Technologies (ONT) sequencing were prepared with the LSK110 Ligation Sequencing Kit (ONT, SQK-LSK110) and loaded onto a R9.4.1 pore PromethION Flow Cell (ONT, FLO-PRO002) (~0.5-1 µg sheared gDNA per flow cell) and run on PromethION 24 sequencer (serial PCA100172) for 72 hours. Data was collected using MinKNOW version 23.04.5. Current signals were converted to DNA bases using Guppy v6.5.7 (ONT) in high-accuracy mode (450 bps), yielding 148.37 Gb pass (of 187.07 Gb estimated, 16.14 M reads; N50 ≈ 14.9 kb; total data written 1.47 TB).

Reads were aligned to hg38 with minimap2 and processed with samtools:

minimap2 -ax map-ont hg38.fa reads.fastq | samtools view -b - | samtools sort -o sample.sorted.bam && samtools index sample.sorted.bam

GpC methylation was called with nanopolish[99]: nanopolish call-methylation -t 48 -q cpggpc -r reads.fastq -b sample.sorted.bam -g hg38.fa > sample.modcalls.tsv

**Motif-centered mapping of ZF single-molecule accessibility**

We defined high-confidence CTCF sites by intersecting CUT&RUN peaks (SEACR, stringent mode) with ENCODE CTCF ChIP–seq peaks (hg38 reference). Summits were called independently for each CUT&RUN replicate; sites present in ≥2 replicates were retained (bedtools multiinter). Consensus summits were expanded ±25 bp and overlapped with ENCODE HEK293 CTCF narrowPeak calls (ENCFF314ZAL).To locate exact motif instances, the JASPAR CTCF PWM (MA0139.1) was used to scan summit-anchored sequences with FIMO[73] ($p < 1\times10^{-4}$). FIMO coordinates (1-based, inclusive) were converted to strand-aware BED entries at motif centers (respecting the N-to-C orientation of the CTCF motif). Motif calls closer than 36 bp center-to-center were removed.

Single-molecule accessibility was derived from nanopolish per-read GpC calls. Nanopolish calls were intersected with the ±1 kb windows surrounding the motif center (bedtools intersect). To avoid positional ambiguity, we retained singleton k-mers (exactly one GpC per k-mer) and assigned per-read states from the nanopolish log-likelihood ratio (LLR): M(methylated) if LLR ≥ 1, U(unmethylated) if LLR ≤ −1, otherwise N (undetermined). The modified base within each k-mer was realigned to the reference by locating the modified position in the k-mer string and projecting it to genome coordinates. The resulting table contained GpC signal within 22,460 CTCF motifs used for the analysis.

For the inaccessibility metaplot (Fig. 3D), we averaged the unmethylated (U) fraction within ±200 bp of the motif center across the 22,460 motifs without excluding reads.

For the ZF accessibility plot (Fig. 3F), the strand-aware offset from the motif center was partitioned into 3-bp bins corresponding approximately to ZF1–ZF11 (per the crystal structures, assuming each ZF interacts with a DNA triplet)[20]. To remove nucleosome reads covering CTCF motif, motif×read pairs were excluded if all GpCs in a flanking nucleosome-depleted region (NDR) window (±20±60) were unmethylated (U). We defined a read as "CTCF-bound" if at least one core ZF bin contained an unmethylated GpC (i.e., protected/inaccessible). Using these reads, we computed the per-motif fraction inaccessible for each ZF.

For the conditional-probability analysis (Fig. 3G), nucleosome-excluded reads were subjected to a molecule-level filter requiring ≥2 distinct core ZFs bound (U) per read and reads with all measured ZFs bound were removed to focus on the dynamic subset. We then calculated conditional probability P(ZFj=1| ZFi=1). ZF5 was excluded due to low coverage.

For the correlation analysis, cohesin subunits ChIP–seq signals (downloaded from 4DN[100] portal[101], currently publicly available in Gene Expression Omnibus (https://www.ncbi.nlm.nih.gov/geo/) with the accession number GSE277721[77]) and ENCODE for REST (dataset: ENCFF981ILL) were extracted from normalized bigWig files as the median signal in a ±200 bp window around each motif center. We computed per-motif, per-bin CTCF-bound ZF "binding probabilities" (fraction of reads with U; numerator = reads with any U in the bin; denominator = measured reads in the bin) restricted to dynamic molecules (≥1 bound and ≥1 unbound core ZF across ZF1–ZF11). Binding probabilities were correlated with per-motif ChIP signals for the following ChIP-seq GSE277721 datasets (REST, STAG2, PDS5A, NIPBL, STAG1, RAD21-NIPBL degron 24h, RAD21, CTCF). For each factor and ZF bin (ZF1–ZF11, excluding ZF5) we computed Pearson's r across motifs with raw p-values and controlled the false discovery rate using Benjamini–Hochberg across all factor×ZF tests. ZF5 was excluded from analysis due to low GpC coverage.

#### *Analytical methods*

##### Unzipping data processing and analysis

Experimental data were digitized at 2,500 Hz, baseline-corrected, and converted into force and extension vectors using calibration parameters. To precisely adjust and subtract any residual extension offset in individual experiments, force-extension curves up to 10

pN were fit to an extensible worm-like-chain (eWLC) model of double-stranded DNA, converting to bps[47,59,60,69], aligning the traces as described previously.[60] The 'distance from the center of the motif' in force-position curves was calculated by subtracting the known position of the ZF6 interaction with the consensus sequence.[19,20]

To calculate the mean breaking force in a specific region, we generated an "interaction vector" for each unzipping trace, where an interaction is defined as an increase in force at constant position, followed by a force drop. The interaction with the highest breaking force inside the boundaries of each region defines the region's breaking force, which is then averaged over the ensemble. The significance of differences in breaking forces was assessed using the K-S test, without assuming normality. Applying the same criteria to data obtained without CTCF resulted in no binding events detected. The higher rupture force population was identified using a Gaussian mixture model and BIC to determine whether a condition was more likely to contain 1 or 2 populations and predict which points fell in each population. 2 populations were more likely for N-terminal WT CTCF+PDS5A (BIC = 525.1 for 1 Gaussian or 455.9 for 2) and WT CTCF+PDS5A+cohesin conditions (BIC = 457.8 for 1 Gaussian or 456.9 for 2).

Force-weighted (FW) dwell time histograms calculated as previously described[46,60,102]. Briefly, the number of data points in 1 bp bins was counted and divided by the sampling rate (2500 Hz), multiplied by the detected rupture force, after subtracting the average background signal of naked DNA.

**Confocal C-Trap measurement analysis**

Data was analyzed using custom python scripts using the Pylake API released by LUMICKS. Kymo tracking feature was used to track the individual traces to obtain the position and time information which was then subsequently used to calculate MSD then diffusion coefficient. Details of which are included in the script submitted in the GitHub.

**AFM image processing and analysis**

HS-AFM images were viewed and analyzed using the software built by Prof. Toshio Ando's laboratory-built software, Kodec 4.4.7.39, with available source. Tilt and other image correction details are available in the literature[103]. The contrast was adjusted to enhance the structural features of the images for visualization.

The bp position of proteins colocalized on the substrate DNA was determined via a semi-automated approach. Firstly, the colocalized proteins were annotated using the circle tool in ImageJ. Hereafter, DNA was annotated by a machine-learning model (manuscript in preparation) and lightly dilated to bridge sub-pixel gaps before skeletonization to a 1-px centerline; edge-touching DNA components were excluded. For each protein ROI, the center was snapped to the nearest skeleton pixel and nearby DNA components within a fixed radius were considered as candidates. The along-contour shortest path from the snap point to the nearest DNA end was then computed on the skeleton graph and skipping circular components lacking endpoints. The candidate yielding the shortest path was selected, and path lengths were converted from pixels to nanometers (using the image-specific pixel size) and then to base pairs using a specified nm/bp calibration for the instrument (manuscript in preparation).

### TIRF analysis

Custom Interactive Data Language (IDL) and MATLAB scripts were used for spot detection and quantification and single-molecule photobleaching analysis. (available at https://sites.google.com/site/taekjiphalab/resources).

### AlphaFold 3 modeling

Predicted AlphaFold 3 structures[51] of CTCF bound to the double-stranded consensus motif, G-rich strand of the consensus motif, C-rich strand of the consensus motif, double stranded *PDGFRA* motif and G-rich strand of the *PDGFRA* motif in the presence of 11 Zn ions were generated on December 5th 2024. These predicted models used as starting configurations of the MD simulations. AlphaFold 3 model confidence was assessed using pLDDT and PAE metrics, as reported in Figure S2, S19, and S24. pLDDT (predicted Local Distance Difference Test) plots were generated by averaging atom pLDDT values for each residue providing a per-residue confidence score reflecting how well each residue's position was predicted. PAE (Predicted aligned error) reports the expected positional error between residue pairs after alignment, indicating the reliability of hypothesized protein-protein interactions.

A predicted structure of PDS5A bound to *PDGFRA*-bound CTCF was generated using AlphaFold 3[51] on April 25th, 2025. Amino acid sequences for CTCF (UniProtKB: P49711) and PDS5A (UniProtKB: Q29RF7) were obtained from UniProt. 11 zinc ions were included in the submitted job. Contact interactions were predicted using PyMOL by identifying inter-chain atom–atom pairs located within 3.5 Å.

### MD simulations

All MD simulations were performed using NAMD2.1[104], 2 fs integration timestep and 2-2-6 multiple time stepping. Bsc1, ffSB14 and ZAFF were used for DNA[105], proteins[106] and ZF[107] respectively, TIP3P for water[108], with CUFIX corrections to model interactions between ions and nucleic acid/proteins[109]. The SETTLE algorithm[110] was employed to enforce rigidity of covalently bonded hydrogen atoms within water molecules. Additionally, for non-water molecules, the RATTLE algorithm[111] was utilized to constrain the motion of hydrogen atoms involved in covalent bonds. The long-range electrostatic interactions were computed using the particle mesh Ewald (PME) scheme over a 1-Å-spaced grid[112]. VDW and short-range electrostatic forces were evaluated using the 10–12 Å smooth cut-off scheme. Non-bonded interactions were implemented using "1-4 scaling" with a scaling factor of 0.833. NPT (constant number of particles *N*, pressure *P* and temperature *T)*

simulations[113] were performed using a Nosé-Hoover Langevin piston with a period of 400 fs, decay of 200 fs at a target pressure of 1 atm and Langevin thermostat[114] set at 298 K with a 0.5 $ps^{-1}$ damping. Simulations performed in the NVT (constant number of particles *N,* volume *V* and temperature *T)* ensemble employed the Langevin thermostat. Energy minimization was carried out using conjugate gradients[115].

Predicted AlphaFold 3 structures were converted to a PDB format using VMD[116] and used as starting configurations. The intrinsically disordered domains of CTCF (residues 1-265 and 585-727) were truncated, and only the zinc finger-DNA complex was simulated. The resulting construct avoids confounding, non-specific interactions from the disordered tails while preserving Zn finger interactions, geometry and topology. The protein was protonated at pH 7 using H++[117]. The PDBs were converted to Amber format, solvated and ionized to produce a neutral system with 0.15 M KCl using the tLEaP package of AmberTools22[118]. Atomic coordinates were saved every 18 ps. VMD[116] and MDAnalysis[119] were used for visualization and analysis. Each system was minimized for 20,000 steps and equilibrated in the NPT ensemble for 4.6 ns. During minimization and NPT equilibration, harmonic restraints with a spring constant of 5 kcal/(mol $Å^2$) were applied to non-hydrogen atoms of nucleic acid and protein. The production simulations (2 replicas of each system), each spanning 1 μs, were performed within an NVT ensemble, devoid of any restraints.

Hydrogen-bond analysis: DNA-protein hydrogen bonds were identified using a donor - acceptor distance cutoff of 3.5 Å and an angle within 30° of linearity (donor–hydrogen–acceptor).

**Dynamic trace preprocessing and formatting**

The dynamic traces were prepared for Hidden Markov Model (HMM) state determination by first cropping the sections containing CTCF binding, followed by linear correction to account for experimental drift. Finally, intercepts were aligned to remove baseline variability, ensuring consistent state identification and improved HMM parameter estimation (see Figure S12). The number of bps unzipped was calculated by subtracting the force-dependent extension of the handles and then dividing by twice the extension of a single ssDNA base at the given tension as described previously[47,52].

**Hidden-state model selection and cross-condition consensus**

All analyses were performed in Python 3.9 using the package hmmlearn (for HMM).

*Model fitting:* Force-time trajectories from individual molecules were first concatenated after removing invalid values. These one-dimensional force series were then modeled with Gaussian-emission HMMs, in which each hidden state is associated with a Gaussian distribution of forces and transitions between states follow a Markov chain. For each candidate number of hidden states (from 1 up to 5), a separate HMM was trained by expectation-maximization (EM). The algorithm iteratively adjusted transition probabilities and emission parameters until the improvement in model fit was smaller than a set tolerance ($10^{-2}$) or a maximum of 300 iterations was reached. For every fitted model, we recorded the overall log-likelihood (how well the model explained the observed data), as well as information-theoretic model selection criteria.

*Model selection criteria:* To decide how many states best described the data, we compared models using two standard information criteria: the Akaike Information Criterion (AIC) and the Bayesian Information Criterion (BIC). Both criteria balance model fit against model complexity, penalizing models with more parameters unless they provide a substantially better fit. In addition, we examined the incremental log-likelihood gain when adding an extra state. This allowed us to identify when increasing the number of states yielded large improvements (as for three to four states) versus when improvements were marginal (as for four to five states).

*Per-condition evaluation and consensus determination:* For each experimental condition, models with up to five states were fitted, and the number of states minimizing the BIC was identified. To obtain a global consensus across conditions, BIC curves were aligned by state number and averaged, and the consensus state number was defined as the one that minimized this mean BIC. In addition, we examined the mean BIC improvement between successive state numbers. Following Kass and Raftery, we defined an “elbow” at the point where the average BIC improvement dropped below 10 units, indicating that adding more states no longer provided meaningful explanatory power (Fig. S5A).

*Application to our dataset:* Applying this approach, eight of nine experimental conditions reached their minimum BIC at four states, including the consensus sequence, *PDGFRA* N-terminal CTCF WT, *PDGFRA* N-terminal CTCF WT with PDS5A, methylated PDGFRA N-terminal CTCF WT, nucleosome-associated C-terminal CTCF WT, *PDGFRA* C-terminal CTCF WT, and *PDGFRA* N-terminal CTCF ΔN. The mean BIC across all conditions was likewise minimized at four states, establishing this as the consensus model size. Only one condition — *PDGFRA* N-terminal CTCF WT + Cohesin — showed a further decrease in BIC when a fifth state was added. The log-likelihood gain from four to five states was modest (~155 units), but still sufficient to exceed the BIC penalty, yielding a ΔBIC of ~136, but the improvement was negligible compared to the large increase observed when moving from three to four states (~83,000 units). We therefore decided to use four states, as that best captures the data across conditions (Fig. S5B).

*Final model fitting:* Once the consensus number of states was determined, we refit the final HMMs for each condition on the full dataset using hmmlearn. Models were initialized with diagonal covariances, quantile-spaced means, and a sticky transition prior (70% self-transition probability). A sticky transition matrix makes the HMM conservative — it only changes state when the data strongly support it. This produces more stable state assignments and avoids overinterpreting noise as new states. Start probabilities were set uniformly, and automatic reinitialization was disabled. Models were trained for up to 1000 EM iterations or until convergence (tolerance $10^{-3}$).

**Transition rate and energy landscape calculations, and visualization**

The free energy landscape is constructed by determining the well heights ($G_i$) and barrier heights ($G_{ij}^{\ddagger}$) from transition probabilities (Fig. S9) and state lifetimes (Table S2) assuming thermal equilibrium[50,120,121]. These calculations rely on equilibrium relationships and transition state theory to estimate the relative free energies of states and activation barriers. To compute the free energy of each state, the equilibrium constant between states $i$ and $j$ is determined from the forward and reverse rate constants, defined in equation 1.

$$K_{ij} = \frac{k_{ij}}{k_{ji}}$$

(equation 1)

Where $k_{ij}$ represents the rate constant for the transition from state $i$ to state $j$, and $k_{ji}$ represents the rate constant for the reverse transition. The rate constants are derived from the transition probabilities ($P_{ij}$) and state lifetimes ($\tau_i$) where the total transition rate out of a state is given by equation 2. Here, the transition probabilities exclude self-transitions / the probability of not transitioning to another state. Errors on the transition rate are calculated from error propagation of $P_{ij}$ and state lifetimes $\tau_i$. Where the error on $P_{ij}$ is calculated as the standard deviation between replicates and the error on $\tau_i$ is estimated as the error from fitting an exponential CDF.

$$k_{ij} = \frac{P_{ij}}{\tau_i}$$

(equation 2)

Using the equilibrium constant, the free energy difference between states $i$ and $j$ is computed as given in equation 3.

$$\Delta G_{ij} = -k_B T ln(K_{ij})$$

(equation 3)

Where $k_B$ is the Boltzmann constant and $T$ is the absolute temperature. The free energy of each state is then calculated iteratively relative to a reference state, typically setting $G_1 = 0$, such that the relation stands as in equation 4.

$$G_j = G_i + \Delta G_{ij}$$

(equation 4)

Barrier heights are computed relative to the well energies by considering the activation free energy required for a transition between states. The forward barrier height is determined from the rate constant as given in equation 5.

$$G_{ij}^{\ddagger} = G_i - k_B T ln\left(\frac{k_{ij}}{\nu_{ij}}\right)$$

(equation 5)

Where $\nu_{ij}$ represents the attempt frequency, an intrinsic property of the system characterizing how often transitions are attempted. Although $\nu_{ij}$ would vary between conditions, as $\nu_{ij}$is unknown, we used the same value for all transitions and conditions, approximated by the Eyring prefactor[122] ($\nu = k_B\, T/h$). Similarly, the reverse barrier height is computed as given in equation 6.

$$G_{ji}^{\ddagger} = G_j - k_B T ln\left(\frac{k_{ji}}{\nu_{ji}}\right)$$

(equation 6)

The energy landscape is visualized by plotting the state energies and transition barriers along a continuous reaction coordinate. All energies are expressed relative to the reference state I, which is set to zero. The energy levels ($G_i$) are positioned at discrete state indices, while the barrier heights ($G_{ji}^{\ddagger}$) are placed at intermediate positions between adjacent states. To ensure a smooth representation of the energy landscape, the energy values are interpolated using piecewise cubic Hermite interpolation (PCHIP). This method preserves the shape of the landscape without introducing artificial oscillations. To facilitate this interpolation, additional artificial data points are introduced at the edges of the landscape with elevated energy values, ensuring a visually consistent upward curvature at the boundaries. The interpolated curve is then evaluated at finely spaced reaction coordinates, generating a continuous trajectory that represents the transition between states.

**Trace categorization for PDS5A and cohesin traces by HMM model selection**

Due to the nature of the optical tweezers experiment, we could not verify which additional proteins were present on CTCF-bound-DNA upon addition of PDS5A or cohesin or PDS5A and cohesin. To categorize how well individual traces correspond to different protein compositions, we constructed HMMs for each experimental condition and compared their ability to explain the observed data. As a common starting point, we used a reference HMM trained on pure CTCF traces, which defined the emission distributions for four conformational states of the DNA-protein complex. The emissions (Gaussian distributions of the measured force signal) were kept fixed across all subsequent analyses to ensure that the state identities remained consistent across conditions. Meaning that state I always represented the highest-force, fully bound state, while state IV represented unbound DNA-like fluctuations.

For each condition beyond CTCF, we adapted the kinetics of the reference model using all available traces for that condition. Each trace was decoded using the Viterbi algorithm, producing a most-likely state sequence. From these sequences, we tabulated the number of times each state was visited first, and the number of observed transitions between every pair of states. These counts were converted into probabilities by normalizing over the total dwell time per state, with a small smoothing factor added to avoid zeros. In this way, we obtained condition-specific estimates of the transition matrix and starting probabilities, while keeping the emission distributions fixed. The result was a new HMM for each condition that retained the same state definitions as the CTCF reference but differed in its dynamical parameters. For stability, these kinetics-adapted models could optionally be further refined by a short round of expectation-maximization, which adjusted only the start and transition probabilities.

Once these models were built, every trace was evaluated by computing its log-likelihood under each of the models deemed biologically plausible for that dataset. For a given observed trajectory $X = \{x_1, \dots, x_T\}$, the HMM likelihood is defined in equation 9.

$$p(X|M) = \sum_{z_1,\dots,z_T} \pi_{z_1} b_{z_i}(x_1) \prod_{t=2}^{T} a_{z_{t-1},z_t} b_{z_t}(x_t)$$

equation 9

Where $\pi$ are the starting probabilities, $a_{ij}$ the transition probabilities, and $b_j(x)$ the Gaussian emission densities for state j. Because the emissions are identical across models, differences in likelihood reflect only how well the observed temporal patterns of switching agree with the condition-specific kinetics. Each trace was assigned to the model that maximized the log-likelihood. In addition, we calculated the difference in log-likelihood between the selected model and the pure CTCF model ($\Delta LL$). This provided a measure of how strongly the trace deviated from CTCF-like behavior and therefore how confidently it could be attributed to the presence of PDS5A, cohesin, or both. Through this procedure, every trace was categorized into the regime that best explained its dynamics, while ensuring that state definitions were preserved across all conditions. This strategy allowed us not only to separate traces into CTCF-only versus factor-bound regimes, but also to quantify how strongly each factor altered the kinetics of DNA-protein interactions. In the Results, we use this framework to show that traces exhibiting factor-specific kinetic signatures can be robustly distinguished from those consistent with CTCF alone.
